# Chemogenetic Minitool for Dissecting the Roles of Protein Phase Separation

**DOI:** 10.1021/acscentsci.3c00251

**Published:** 2023-07-07

**Authors:** Chan-I Chung, Junjiao Yang, Xiaokun Shu

**Affiliations:** 1 Department of Pharmaceutical Chemistry, University of California—San Francisco, San Francisco, California 94158, United States; 2 Cardiovascular Research Institute, University of California—San Francisco, San Francisco, California 94158, United States; 3 Helen Diller Family Comprehensive Cancer Center, University of California—San Francisco, San Francisco, California 94158, United States

## Abstract

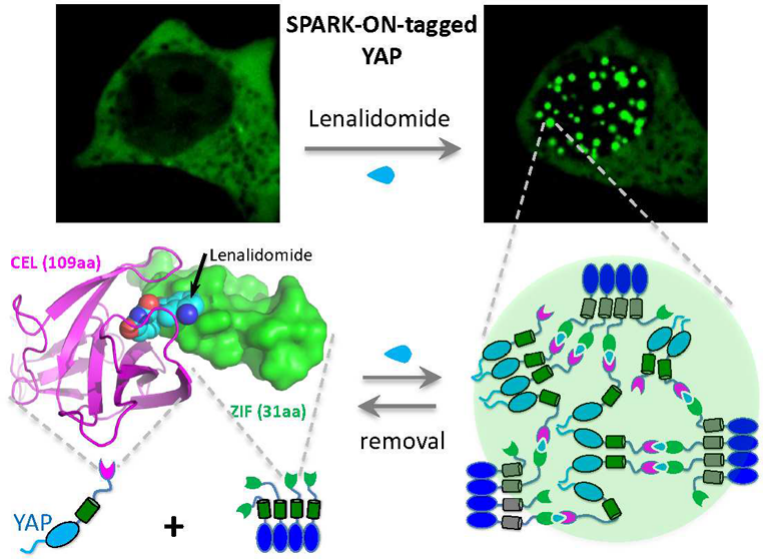

Biomolecular condensate is an emerging structural entity that regulates various cellular processes. Recent studies have discovered the phase-separation (PS) capability of several transcription factors (TFs) including YAP/TAZ upon biological stimuli, which provide new mechanisms of gene regulation. However, it remains mostly unanswered as to whether PS from a diffuse state to a phase-separated state promotes gene transcription. To address this question, we have designed a chemogenetic tool, dubbed SPARK-ON, which manipulates the PS of YAP and TAZ without a biological stimulus, forming condensates that are transcriptionally active, containing the DNA-binding partner TEAD, genomic DNA, transcriptional machinery, and nascent RNA. Most importantly, PS of TAZ increases the transcription of its target genes. Therefore, our data indicate that PS promotes gene transcription of TAZ. SPARK-ON is advantageous to current mutagenesis-based approaches that are often problematic when mutagenesis affects the transcriptional activity of a TF. Furthermore, protein abundance levels also affect gene transcription, but PS depends on protein abundance because PS occurs only when the protein level is above a saturation concentration. SPARK-ON decouples PS from protein abundance levels without introducing mutations and thus will find important applications in understanding the biological roles of PS for many TFs and other biomolecular condensates.

## Abstract 2

Chemogenetic tool SPARK-ON decouples phase separation from protein levels, drives transcription factor condensate formation, and enables dissection of the biological role of phase separation.

## Introduction

Biomolecular condensates are important biological structures that play key roles in multiple cellular processes.^1^ Many of them are membraneless organelles. Two major breakthroughs in the fundamental understanding of these structures have revealed that biocondensates (e.g., P granules) form via phase separation (PS).^2^ PS is often driven by multivalent interactions.^3^ Multivalence is introduced by folded multidomains in a protein or single folded domains that form oligomeric complexes as well as by intrinsically disordered regions (IDRs) that contain multiple charged or aromatic residues mediating weak and multivalent interactions or via the stickers-and-spacers model.^4^ Functional studies indicate that many condensates are biologically active such as in cell signaling and gene transcription.^1,5−10^ For example, recent studies of several TFs show that they undergo phase separation (PS) and form biomolecular condensates,^11−17^ including transcriptional effectors of the Hippo pathway, YAP and TAZ, when the concentration of the proteins surpasses a threshold caused by an environmental stimulus such as osmotic stress for YAP.^18^ These TF condensates are further shown to be transcriptionally active.^1,5−9^ For example, the YAP and TAZ condensates contain transcriptional machinery.^18−20^

Although many TF condensates contain transcriptional machinery,^11−14,17^ whether phase separation really changes the transcriptional output is still controversial.^21^ Answers to this key question are hampered by conceptual and technical challenges. Several studies introduced mutations to change the phase behavior in order to correlate the driving force for phase separation with transcription.^13,18^ These mutations are often introduced into the TF’s activation domain that harbors the PS-promoting IDRs, but the activation domains often interact with the mediator that loops enhancer to promoter via interaction with RNA polymerase II and general transcription factors.^22^ Thus, the mutations that are introduced into the activation domain in order to block PS will likely also impact the ability of TFs to form diffuse complexes with transcriptional machinery.^12,23^ Unfortunately, many previous studies reached conclusions that phase separation-activated transcription is based on the PS-blocking mutations that reduce transcription without evidence that the introduced mutations do not affect the interaction of the TFs with the mediator and transcriptional machinery.^12,13,18,23^ This suggests that the reduction in transcriptional output can be due to the blocked PS and/or the reduced interaction with the transcriptional machinery. Therefore, it is still under debate if the transcriptional output in the presence of TF condensates would also be achieved in the absence of phase separation by diffuse complexes. Determining the role of phase separation in transcription is thus much needed. The conceptual and technical challenges call for new tools that enable us to assess transcriptional activity upon dissolving condensates without introducing mutations or changing expression levels.

Enabling technologies, that are capable of manipulating PS without changing protein levels or introducing mutations, can help us gain a mechanistic understanding and appreciation of the functional roles of phase separation. These technologies include optogenetic tools such as the Cry2-based OptoDroplet and the ferritin/iLID/sspB-based Corelet^54,56,59−62^ and chemogenetic tools such as the FKBP/Frb-based systems as well as the eDHFR/HaloTag-based tools.^29,24,57,63,64^ Such tools enable us to decouple the role of phase separation from the change in protein expression levels. Optogenetic tools achieve the subcellular manipulation of protein phase separation and are very valuable in understanding biological condensates.^54,56^ On the other hand, one advantage of chemogenetic tools is that a small-molecule-based approach simplifies sample processing with a large number of cells for biochemical characterization including genetic analysis such as RT-qPCR. While chemogenetic tools such as the rapamycin-inducible FKBP-Frb system have been used in manipulating PS,^29,57^ they have not been applied to the phase separation of transcription factors, and the rapamycin-mediated FKBP-Frb interaction is stronger than those in the biomolecular condensates that mostly form via weak interactions.

Here, we decided to engineer a new chemogenetic tool that is capable of manipulating PS and contains several unique capabilities: (1) it uses FDA-approved drug molecules; (2) the PPI pair is smaller than the FKBP-Frb pair, with one component as small as ∼30-aa; (3) the interaction is weak so that it better mimics the weak interaction in most of the biomolecular condensates; and (4) the drug-induced weak interaction allows the quantitative manipulation of PS and thus enables us to determine role of PS in a quantitative manner. Such versatile tools enable us to decouple the role of phase separation from a protein expression level-induced or mutation-induced change in protein activity. Furthermore, small molecule-activatable chemogenetic tools that are compatible with genomic analysis approaches will facilitate functional characterizations such as the profiling transcription of specific genes. Such chemogenetic tools are powerful technologies in determining the role of phase separation in gene regulation.

To engineer such versatile chemogenetic tools, we first designed a small molecule-inducible protein heterodimer. Then we introduced *de novo* designed multivalent tags into the heterodimer to induce multivalent PPIs that drive PS. This small molecule-activatable chemogenetic tool is capable of driving PS and forming liquid-like condensates that are biologically active. The engineered chemogenetic tools are compatible with genetic analysis approaches including RT-qPCR.

## Results

### Structure-Based Design of the IMiD-Inducible Protein Heterodimer for Controlling PPI

**Figure 1:**
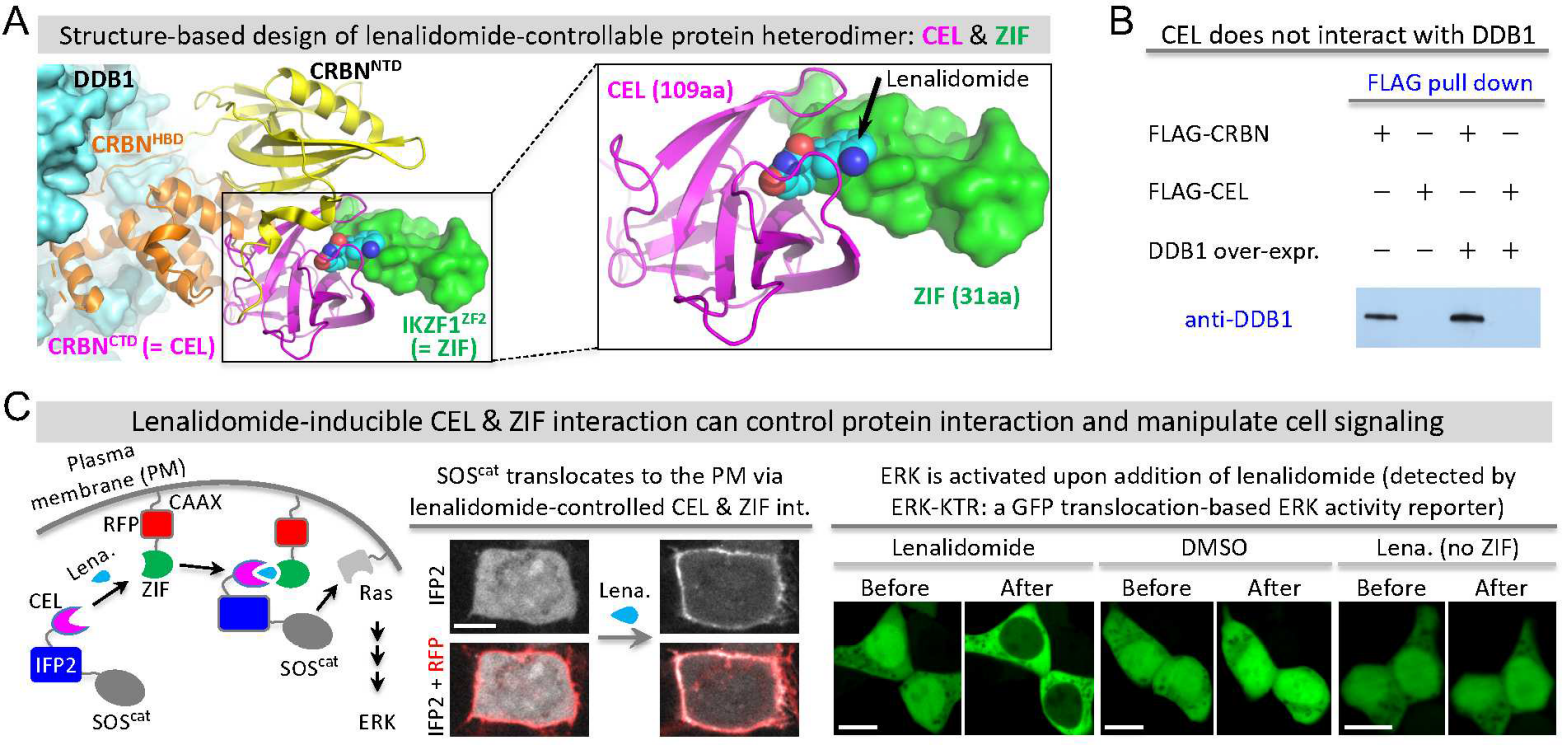
Structure-based design of the lenalidomide-inducible protein heterodimer for controlling protein–protein interactions. (A) Structural model of the lenalidomide-induced protein complex containing DDB1, cereblon (CRBN), and zinc finger 2 (ZF2) of Ikaros (IKZF1), built by SWISS model using the crystal structure of DDB1-CRBN-CK1α (pdb: 5fqd). The model illustrates the design of lenalidomide-controllable protein heterodimers CEL (i.e., CBRN^CTD^) and ZIF (i.e., IKZF1^ZF2^). CRBN^CTD^: c-terminal domain of CRBN. IKZF1^ZF2^: zinc finger 2 of IKZF1. (B) Western blot against DDB1 after FLAG pull-down of CRBN or CEL. HEK293 cells were transfected with FLAG-tagged CRBN or CEL in the absence or presence of exogenous DDB1 overexpression. (C) Left: schematic showing the translocation of SOScat from the cytoplasm to the plasma membrane via the lenalidomide-induced interaction between CEL and ZIF. The relocated SOScat then activates Ras, which leads to ERK activation via the MAPK pathway. Middle: fluorescent images upon addition of 1 μM lenalidomide to HEK293 cells expressing CEL-IFP2-SOScat and ZIF-RFP-CAAX. See Movie S1. Right: fluorescent images of HEK293 cells expressing ERK activity reporters ERK-KTR, CEL-IFP2-SOScat, and ZIF-RFP-CAAX upon addition of 1 μM lenalidomide. Scale, 10 μm.

To engineer a chemogenetic tool that can induce condensate formation, we turned to the immunomodulatory drug (IMiD)-inducible PPI pair of cereblon (CRBN) and Ikaros (IKZF1) because our previous study showed that IMiDs such as lenalidomide can induce condensate formation when the protein pair is tagged with multivalent tags.^24^ IMiDs, including thalidomide and lenalidomide, are FDA-approved drugs against multiple myeloma and have no or little toxicity in other cells.^25^ IMiD-dependent interactions between CRBN and IKZF1 bring the transcription factor IKZF1 to the cullin ring E3 ubiquitin ligase complex CUL4-RBX1-DDB1-CRBN via the interaction between CRBN and the adaptor protein DDB1, resulting in ubiquitination and degradation of IKZF1.^26^ To design a stable IMiD-controllable protein heterodimer, we sought to disrupt the interaction between CRBN and DDB1. Structural studies of DDB1-CRBN-IKZF1 show that the helical bundle domain (HBD) of CRBN interacts with DDB1 but does not bind lenalidomide or interact with IKZF1 (Figure 1A).^27^ On the other hand, the C-terminal domain (CTD) of CRBN binds lenalidomide and interacts with IKZF1, without direct contact with DDB1. Additionally, the N-terminal domain (NTD) of CRBN does not appear to interact directly with DDB1 or IKZF1. Therefore, we first truncated the NTD and HBD of CRBN but retained the 109 amino acid (aa) CBRN CTD, which was renamed CEL. Next, we truncated IKZF1 so that only the zinc finger 2 (ZF2) domain was retained because ZF2 binds lenalidomide and interacts with CEL (Figure 1A).^27^ The ZF2 of IKZF1 contains 31aa and is referred to as ZIF (Figure 1A). We verified that CEL does not interact with endogenous DDB1 (or exogenous DDB1) based on immunoprecipitation, whereas CRBN interacts with DDB1 (Figure 1B).

To demonstrate that the engineered CEL and ZIF can be used to control PPI via lenalidomide, we tested whether this system can drive subcellular translocation of the GTP exchange factor SOS to the plasma membrane (PM). The presence of SOS on the PM can activate Ras and promote ERK activity via the MAPK pathway (Figure 1C, left). For targeting SOS to the PM, we first fused ZIF to the CAAX motif tagged with a red fluorescent protein (RFP). Next, we fused CEL to the catalytic domain of SOS (referred to as SOScat), which was tagged with near-infrared fluorescent protein IFP2 that helps visualize SOScat translocation (Figure 1C, left). Upon addition of lenalidomide, SOScat translocated from the cytoplasm to the PM (Figure 1C, middle) within 3–5 min (Movie S1). SOScat continued to accumulate at PM at later time points, and membrane ruffling became visible (Movie S1). We also confirmed that the total protein level of SOScat did not change during this process upon addition of lenalidomide (Supporting Figure S3). To determine whether SOScat translocation activates ERK, we used a GFP translocation-based ERK activity reporter called ERK-KTR, which is translocated from the nucleus to the cytoplasm upon activation of endogenous ERK.^28^ Upon addition of lenalidomide, ERK-KTR translocated from the nucleus to the cytoplasm (Figure 1C, right). In contrast, DMSO did not induce any ERK-KTR translocation, and lenalidomide alone did not induce ERK-KTR translocation in cells expressing the CEL-IFP2-SOScat without ZIF. We further verified ERK activation upon lenalidomide-induced SOScat translocation using another ERK activity reporter ERK-SPARK (Supporting Figure S1, Movies S2–S4).^29^ Thus, we have demonstrated that the lenalidomide-inducible CEL and ZIF heterodimer can be used to control PPI and cell signaling.

### Multivalent PPI-Based Chemogenetic Tools for Manipulating Protein Phase Separation

**Figure 2:**
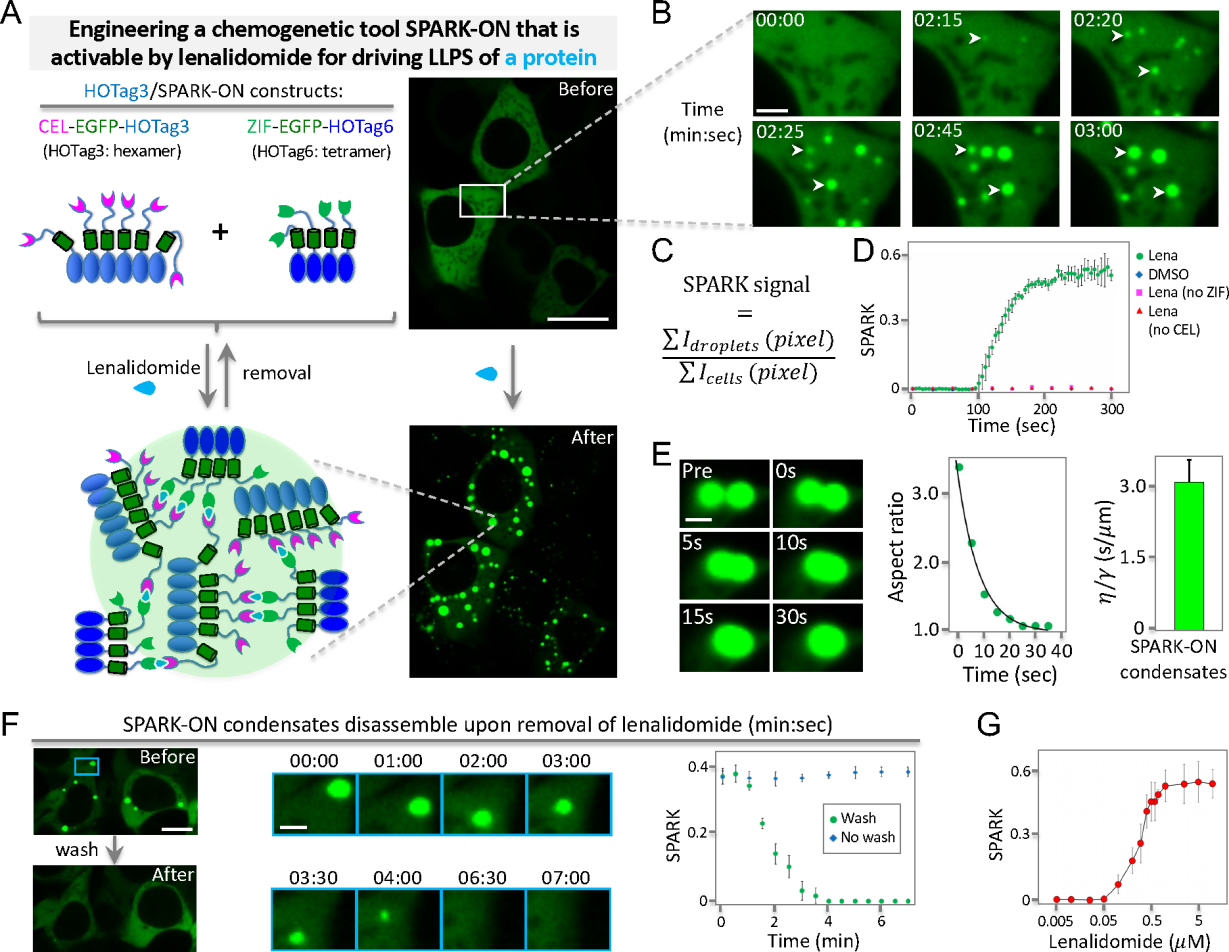
Engineering a lenalidomide-activatable chemogenetic tool for manipulating protein phase separation. (A) Left: schematic of lenalidomide-activatable chemogenetic tool SPARK-ON for controlling the PS of a protein of interest (POI). POI/SPARK-ON constructs are CEL-EGFP-POI and ZIF-EGFP-HOTag6. Here POI is HOTag3. POI can also be YAP or other proteins. Right: fluorescent images before and after the addition of lenalidomide to HEK293 cells expressing the two constructs shown on the left. (B) Time-lapse images corresponding to the boxed area in (A). (C) Definition of the SPARK signal, which is the ratio of total droplet fluorescence over total cellular fluorescence. The fluorescence intensity of all droplets is summarized from each pixel of droplets. The total cellular fluorescence is summarized from all fluorescent pixels. (D) Quantitative analysis of droplet formation over time after the addition of lenalidomide or DMSO in HEK293 cells expressing HOTag3/SPARK-ON or without CEL or ZIF. The error bar represents the standard deviation (*n* = 3). (E) Left panel: Time-lapse images showing the fusion events of two condensates in HEK293 cells. Middle panel: Aspect ratio of two fusing droplets over time. Right panel: Inverse capillary velocity averaged from seven fusion events. The error bar represents the standard deviation (*n* = 7). (F) Time-lapse images showing droplet disassembly after the removal of lenalidomide. HEK293 cells were preincubated with lenalidomide for 10 min. Time-lapse imaging started after lenalidomide removal. Time is in min:sec. The error bar represents the standard deviation (*n* = 3). (G) Titration curve of the normalized SPARK signal in cells incubated with various concentrations of lenalidomide. The error bar represents the standard deviation (*n* = 3). Scale bars: (A) 20 μm, (B) 3 μm, (E) 1 μm, (F) 10 μm, and (F) inset: 2 μm.

To engineer the CEL/ZIF system into a chemogenetic tool that can control protein PS, we introduced multivalency into the lenalidomide-dependent PPI system because multivalent PPI can drive protein phase separation.^3^ We fused CEL and ZIF to the homo-oligomeric tags (HOTag) that are *de novo* designed coiled coils: CEL to HOTag3 (30 aa) and ZIF to HOTag6 (33 aa) (Figure 2A, Supporting Figure S2). HOTag3 and HOTag6 have previously been characterized as a hexamer and tetramer, respectively.^29−31^ To visualize phase separation, we tagged both constructs with the enhanced GFP (EGFP) (Figure 2A) and used time-lapse imaging to visualize the phase separation, growth, and fusion of protein droplets (Figure 2B–E). The addition of lenalidomide induced EGFP phase separation and formed bright fluorescent droplets, suggesting that lenalidomide-dependent and HOTag-based multivalent PPI between CEL and ZIF leads to protein phase separation (Figure 2A). At first, small protein droplets formed (∼200–400 nm in diameter at 2 min 15 s after the addition of lenalidomide), which rapidly grew into medium-size droplets, and these droplets continued to grow into relatively large droplets (∼1.5 μm at 3 min) (Figure 2B, Movie S5). We named this technology SPARK-ON (Separation of Protein phases Activatable and Reversible by small molecule-based Kinetic control). For proteins that can form condensates, they often contain a multivalent domain or an IDR. The SPARK-ON chemogenetic tool is engineered to drive the PS of such a protein of interest (POI) by lenalidomide-inducible multivalent interactions. SPARK-ON-tagged POI is referred to as POI/SPARK-ON, which is composed of two constructs: CEL-EGFP-POI and ZIF-EGFP-HOTag6. In the above demonstrated case, POI is HOTag3. As shown below, POI can also be a protein that has a tendency to form condensates, such as a TF (e.g., TAZ nad YAP).

We quantified the time-lapse images by calculating the SPARK signal, which is defined as the ratio of the summarized pixel intensity of droplets divided by the summary of the pixel intensity of the total cellular fluorescence (Figure 2C). Quantitative analysis of the time-lapse fluorescent images revealed fast kinetics (within 2 to 3 min) of lenalidomide-induced HOTag3/SPARK-ON droplet formation (Figure 2D). Control experiments showed that DMSO did not induce condensate formation and that lenalidomide alone (lack of ZIF or CEL) could not induce protein phase separation.

We next determined that these droplets have liquid-like properties. We conducted time-lapse imaging and characterized fusion events between two droplets. Droplets fused and coalesced within a few seconds with their total volume conserved: two droplets with 2.4 μm diameter fused into one droplet with 3 μm diameter (Figure 2E, Movie S6). The fusing droplets initially formed a dumbbell shape, which over time relaxed to a spherical shape (Figure 2E, left panels). Quantitative analysis of the two fusing droplets showed that over time the aspect ratio fitted a single exponential curve (Figure 2E, middle panel), which is an established property of coalescing liquid droplets.^32,33^ We characterized seven fusion events and determined the averaged inverse capillary velocity (= η/γ; here γ is the surface tension of the droplet and η is viscosity) to be 3.07 ± 0.46 (s/μm) (Figure 2E, right panel). Thus, quantitative analysis of the fusion events indicates that these micrometer-sized structures are liquid droplets.

We further determined that upon removal of lenalidomide the droplets disassembled within 5 min (Figure 2F, Movie S7). The disassembly process was quantified by calculating the SPARK signal. The reversibility of these HOTag3/SPARK-ON droplets is consistent with the above characterization that they are liquid droplets. Solid-like condensates are known to be irreversible. Finally, the titration of lenalidomide in the cells showed that the degree of droplet formation (measured by the SPARK signal) was dependent on the concentration of lenalidomide with a half-to-maximum value of ∼0.3 μM (Figure 2G).

### Phase Separation of Scaffold Protein G3BP1 Recruits Clients but Not Vice Versa

We next applied SPARK-ON to manipulate biomolecular condensates in the cytoplasm to understand their assembly. To elucidate the assembly process of condensate formation, the composition of biomolecular condensates has been proposed to contain two types of macromolecules: scaffolds and clients.^1,34,35^ The scaffold proteins often contain a domain with a large number of interaction valences, such as an oligomeric domain or an IDR, which is largely responsible for driving phase separation.^36^ A leading model of condensate assembly is that condensates form by the phase separation of scaffold proteins, which subsequently recruits clients that contain a small number of interaction valences.^34^ While this scaffold–client model might be simplified, it greatly helps in understanding condensate assembly.^8,9^

To examine this scaffold–client model and improve our understanding of condensate assembly and composition, we tested whether the phase separation of a scaffold protein itself (without biological stimuli for condensate formation) would recruit client proteins and whether the phase separation of a client protein would recruit scaffold proteins. We applied the SPARK-ON technology to proteins of stress granules (SG). Recent studies have revealed that G3BP1 plays a central role in SG assembly,^37−39^ and G3BP1 has been described as a scaffold protein for SG formation.^34,40^ The client proteins of SG include RNA-binding proteins FUS and TIA-1.^34^

**Figure 3:**
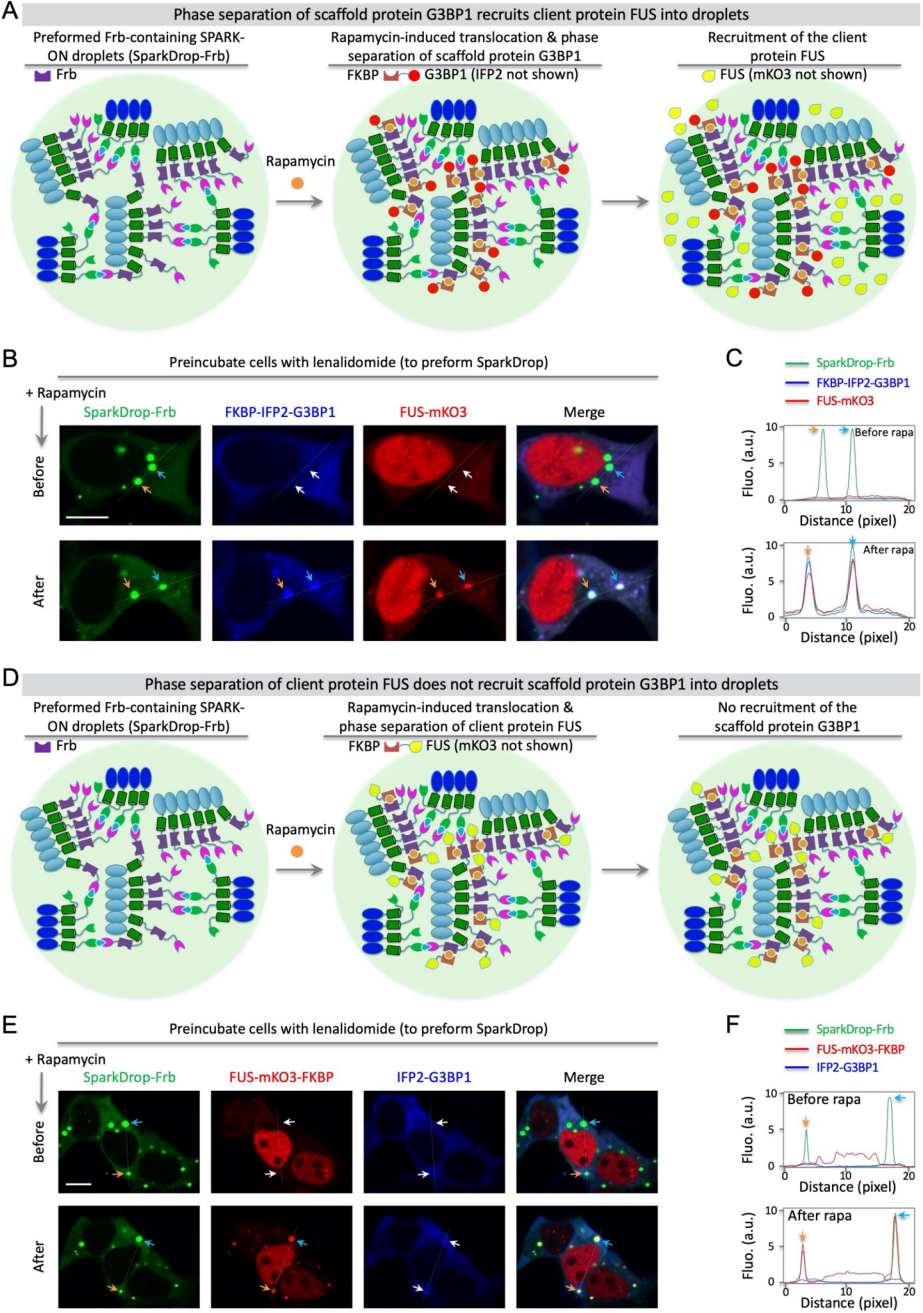
Phase separation of SG scaffold protein G3BP1 can recruit client proteins but not vice versa. (A–C). SPARK-ON-induced (no stress stimuli such as arsenite) phase separation of G3BP1 recruits FUS. (A) Schematic of the experimental design with observed results. (B) Time-lapse fluorescent images before and after (30 min) the addition of 30 nM rapamycin to HEK293 cells expressing SparkDrop-Frb (i.e., constructs of CEL-Frb-EGFP-HOTag3 and ZIF-EGFP-HOTag6), FKBP-IFP2-G3BP1, and FUS-mKO3. (C) Fluorescence intensity plot against distance (dashed lines in panel B). The cells were preincubated with 1 μM lenalidomide for 30 min. (D–F). SPARK-ON-induced (no stress stimuli such as arsenite) phase separation of FUS does not recruit G3BP1. (D) Schematic of the experimental design with observed results. (E) Time-lapse fluorescent images before and after (30 min) the addition of 30 nM rapamycin to HEK293 cells expressing SparkDrop-Frb (i.e., constructs of CEL-Frb-EGFP-HOTag3 and ZIF-EGFP-HOTag6), FUS-mKO3-FKBP, and IFP2-G3BP1. (F) Fluorescence intensity plot against distance (dashed lines in panel E). The cells were preincubated with 1 μM lenalidomide for 30 min. Scale bars: B and E, 10 μm.

We first determined whether SPARK-ON-induced G3BP1 condensates could recruit FUS and TIA-1 in living cells (without applying a stress stimulus such as arsenite), and then we examined whether FUS or TIA-1 phase separation could recruit G3BP1. We combined a lenalidomide-inducible SPARK-ON droplet (SparkDrop) with the rapamycin-inducible FKBP and Frb heterodimer. We incorporated Frb into SparkDrop (referred to as SparkDrop-Frb), fused full-length G3BP1 to FKBP tagged with IFP2, and also tagged FUS with a red fluorescent protein mKO3 (Figure 3A, Methods). First, we preformed SparkDrop-Frb by incubating the transfected cells with lenalidomide (Figure 3B). Then, we added rapamycin to induce FKBP and Frb interaction, which should drive the G3BP1 fusion protein into preformed droplets, resulting in the formation of near-infrared fluorescent droplets. As shown in Figure 3B,C, we observed near-infrared G3BP1 droplets ∼2 min after the addition of rapamycin (Movie S8). Furthermore, red fluorescent FUS droplets were observed after the formation of G3BP1 droplets, which colocalized with both near-infrared and green droplets (Figure 3B,C, Movie S8). And the total protein levels of G3BP1 and FUS underwent no or little change during these procedures (Supporting Figure S4). In control experiments, rapamycin alone (lack of FKBP or Frb) did not induce G3BP1 phase separation or subsequent FUS recruitment (Supporting Figure S5). Thus, these imaging studies indicate that the phase separation of SG scaffold protein G3BP1 recruits the SG client protein FUS.

To induce phase separation of the client protein FUS and determine whether G3BP1 is recruited, we fused FKBP to FUS tagged with mKO3 and fused G3BP1 to IFP2 (Figure 3D). We preformed droplets of SparkDrop-Frb by incubating the transfected cells with lenalidomide, expecting that the subsequent addition of rapamycin should induce FKBP and Frb interaction and drive FUS fusion protein into the preformed droplets to undergo phase separation. However, phase separation of FUS, based on the scaffold–client model, will unlikely recruit the scaffold protein G3BP1 (Figure 3D). Time-lapse imaging revealed that rapamycin induced red fluorescent FUS droplets that colocalized with the preformed green droplets (Figure 3E,F). However, no near-infrared fluorescent droplets were observed, indicating that G3BP1 was not recruited into the FUS droplets. Here we also confirmed that the total protein levels of FUS and G3BP1 had no or little change during the above procedures (Supporting Figure S6). In control experiments, rapamycin alone (lack of FKBP or Frb) did not induce FUS phase separation (Supporting Figure S7). Thus, the imaging studies show that phase separation of the SG client protein FUS does not recruit the SG scaffold protein G3BP1.

We also conducted similar experiments for another SG client protein TIA-1, which showed similar results that SparkDrop-induced G3BP1 condensates recruit TIA-1 (Supporting Figure S8A–C), and rapamycin alone (without FKBP or Frb) does not recruit TIA-1 (Supporting Figure S9). On the other hand, SparkDrop-induced TIA-1 condensates do not recruit G3BP1 (Supporting Figure S8D,E), and rapamycin alone (lack of FKBP or Frb) did not induce TIA-1 phase separation (Supporting Figure S10). Taken together, our data indicates that the SparkDrop-induced phase separation of the SG scaffold protein G3BP1 can recruit the SG client proteins FUS and TIA-1 but not vice versa. Recently, many SG proteins that interact with G3BP1 have been identified,^41^ and the SPARK-ON-based tools will be useful to further characterize these proteins for a mechanistic understanding of SG formation and composition.

### SPARK-ON Enables the Formation of YAP Condensates without Osmotic Stress

To demonstrate whether the SPARK-ON technology can be used to manipulate the condensate formation of a transcriptional factor for dissecting functional roles in gene transcription, we applied it to control YAP condensate formation in living cells. YAP and TAZ are transcriptional coactivators in the Hippo pathway, a highly conserved signaling pathway from *Drosophila* to mammals.^42,43^ They shuttle between the cytoplasm and the nucleus in response to diverse intracellular and extracellular cues including cell–cell contact and hyperosmolarity.^44^ Upon activation, YAP and TAZ are translocated to the nucleus and regulate gene transcription by interacting with the DNA-binding TEAD family transcriptional factors. YAP and TAZ are thus key effectors in the Hippo pathway and play critical roles in animal development and tissue homeostasis.^42,43,45^ Their dysregulation is associated with a plethora of human cancers and is involved in cancer drug resistance.^46,47^ Recently, it was discovered that YAP and TAZ form condensates in the nucleus via their IDRs.^18,48^

**Figure 4:**
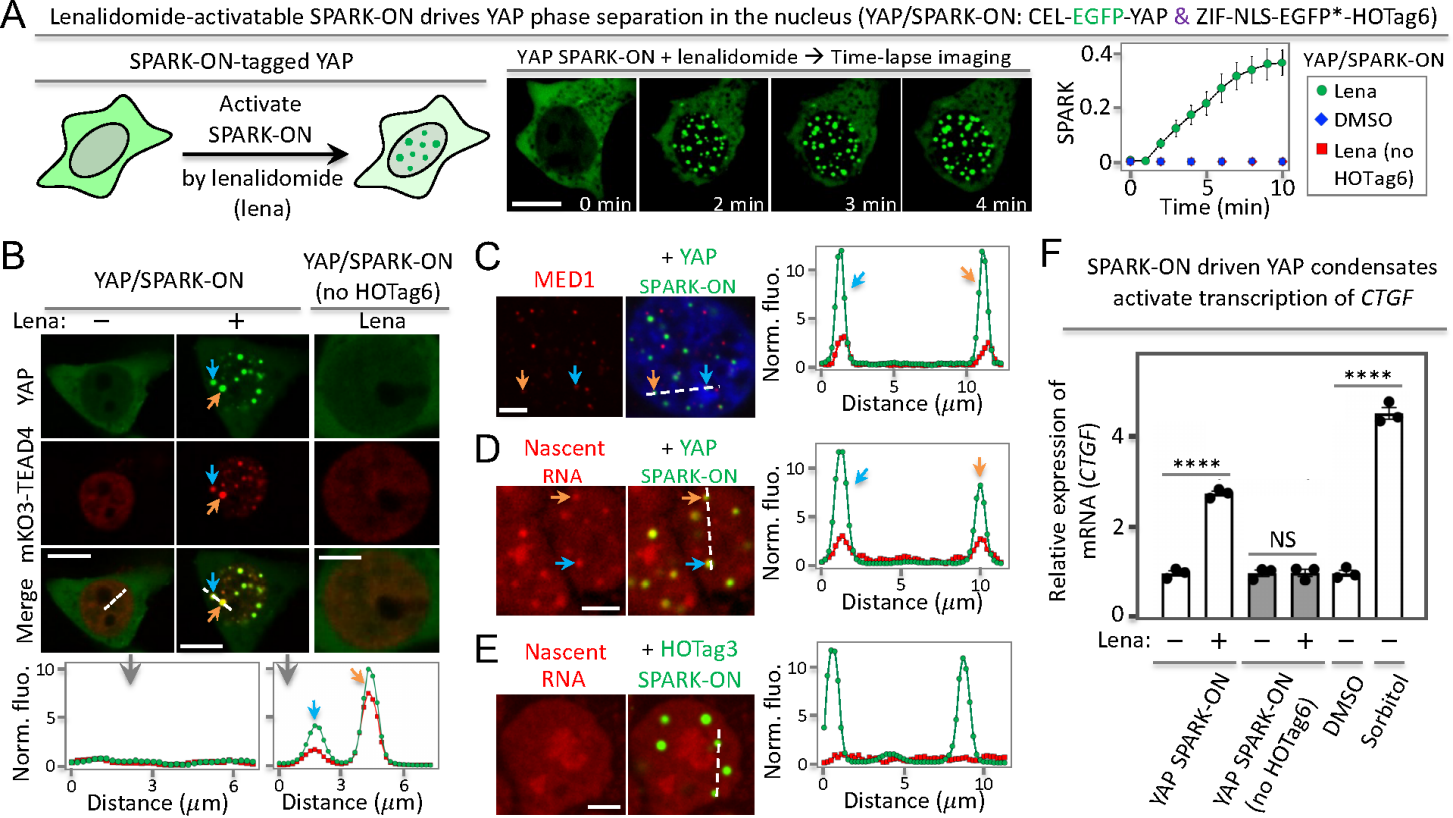
SPARK-ON drives YAP phase separation forming transcriptionally active condensates. (A) Lenalidomide-activatable SPARK-ON induces YAP condensate formation in the nucleus. (B – E). SPARK-ON-driven YAP condensates are transcriptionally active, containing a DNA-binding partner TEAD (B), a mediator (C), and nascent RNA (D). (E) Control HOTag3/SPARK-ON condensates without YAP contain no nascent RNA. Here YAP is replaced by a multivalent tag HOTag3 in order to form condensates upon addition of lenalidomide. (F) RT-qPCR analysis of YAP-target gene *CTGF* upon the SPARK-ON-induced phase separation of YAP. Data are the mean ± SD (*n* = 3). ****P < 0.0001. NS, not significant. Scale bars: 10 μm (A, B – left and middle panels); 5 μm (B – right panels, C–E).

To manipulate the phase separation of YAP without osmotic stress, we applied SPARK-ON to label and control YAP condensate formation without sorbitol stimulation (Figure 4A). We fused YAP to CEL and EGFP, and the fusion protein was localized to the cytoplasm as expected for YAP in the inactive state. We coexpressed ZIF-NLS-EGFP(Y66F)-HOTag6 in the nucleus by incorporating a nuclear localization sequence (NLS). Because lenalidomide induces CEL and ZIF interaction, it is expected that lenalidomide will activate SPARK-ON so that YAP is translocated to the nucleus and forms condensates via multivalent interactions. Indeed, after the addition of lenalidomide, green fluorescent droplets quickly formed in the nucleus at ∼2 min, which grew larger, forming intense green droplets within 10 min. This indicates that SPARK-ON can indeed manipulate YAP phase separation, forming YAP/SPARK-ON condensates (Figure 4A). Quantitative analysis of the time-lapse fluorescent images revealed the kinetics of YAP condensation with a half-maximal time value (*T*_1/2_) of ∼5 min (Figure 4A). We further confirmed that the total protein levels of YAP underwent little change (Supporting Figure S11). As a control, we showed that DMSO did not induce YAP condensate formation. Furthermore, lenalidomide alone could not induce YAP phase separation, using the YAP/SPARK-ON control (without HOTag6) (Figure 4A).

Next, we determined that the SPARK-ON-induced YAP condensates interact and colocalize with the TEAD transcriptional factors using red fluorescent protein mKO3-labeled TEAD4. Fluorescence imaging showed that, before the addition of lenalidomide, YAP was barely detected in the nucleus, whereas TEAD4 was in the nucleus but no TEAD4 was condensed (Figure 4B). After the addition of lenalidomide, YAP formed green condensates in the nucleus and TEAD4 partitioned into the YAP condensates. Quantitative analysis showed that the total protein levels of YAP and TEAD4 underwent little change (Supporting Figure S12). As a control, lenalidomide itself did not induce the condensate formation of YAP. These data indicate that the YAP condensates compartmentalize DNA-binding partner TEAD4 and have the potential to activate gene transcription.

### SPARK-ON-Induced YAP Condensates Activate Gene Transcription

We first determined that the SPARK-ON-induced YAP condensates recruited transcriptional machinery. Because the mediator complex is required for gene transcription by RNA polymerase II (RNAPII), we first examined whether the SPARK-ON-induced YAP condensates recruit MED1 (mediator of RNA polymerase II transcription subunit 1). Immunofluorescence imaging showed that these YAP condensates did colocalize with MED1 condensates (Figure 4C). Some YAP/SPARK-ON condensates did not merge well with MED1, which is likely because some condensates might not yet recruit MED1 and/or the interaction with MED1 could be dynamic, involving the association and dissociation of the two proteins, and some MED1 were likely recruited to the condensates of other transcription factors^13,16^ so that some MED1 puncta did not contain YAP/SPARK-ON.

Next, we determined that the SPARK-ON-induced YAP condensates contained nascent RNA. Here we labeled nascent RNA by incubating the cells with uridine analog 5-ethynyluridine (EU) for 1 h so that EU is incorporated into newly transcribed RNA. The EU-labeled nascent RNA is detected by using a copper (I)-catalyzed cycloaddition reaction (i.e., “click” chemistry) with azides labeled with red fluorescent dyes. Fluorescence imaging in the red channel revealed several punctate structures (Figure 4D). The small and round structures of nascent RNAs were colocalized with the YAP condensates, suggesting that these YAP condensates contain nascent RNAs. Large structures of nascent RNAs were also observed and colocalized with nucleoli (Supporting Figure S13), sites of abundant rRNA transcription, reported to be stained intensely with 5-EU.^49^ As a control, the HOTag3/SPARK-ON droplets without YAP did not contain nascent RNA (Figure 4E). These HOTag3/SPARK-ON condensates did not recruit YAP or TEAD4 (Supporting Figure S14).

Finally, we determined that the SPARK-ON-induced YAP condensates are transcriptionally active and upregulated mRNA levels of a core YAP target gene *CTGF* using reverse transcription quantitative real-time PCR (RT-qPCR). Here we expressed YAP/SPARK-ON, incubated the cells with lenalidomide that activated SPARK-ON, and induced YAP condensate formation. As a control, we incubated cells with DMSO. Our data showed that the YAP condensates upregulated *CTGF* mRNA by ∼2.8-fold relative to that of the DMSO control (Figure 4F). We verified that lenalidomide itself did not change *CTGF* mRNA, using the YAP/SPARK-ON control (no HOTag6). Furthermore, the lenalidomide-activatable SPARK-ON was able to control YAP activity in a quantitative manner in regulating the expression of the target gene *CTGF* by applying different levels of lenalidomide (Supporting Figure S15). As a positive control, we incubated cells with sorbitol, which is known to induce osmotic stress and drive YAP condensate formation. This upregulated the *CTGF* mRNA level relative to that of the untreated cells. Taken together, our results demonstrate that the lenalidomide-activatable SPARK-ON can manipulate YAP phase separation and the induced condensates are transcriptionally active, containing DNA-binding partner TEAD, transcriptional machinery MED1, and nascent RNAs and upregulating YAP target gene transcription.

### Phase Separation of YAP Promotes Its Transcriptional Activity

While a previous study concluded that YAP phase separation played a role in gene transcription, it used a problematic approach by deleting the activation domain,^18^ here we used SPARK-ON to examine role of YAP phase separation in gene transcription. We engineered nuclear localized YAP (nlsYAP) with SPARK-ON. This nlsYAP/SPARK-ON would maintain protein level of YAP in the nucleus, which can enable us to decouple role of PS from change of protein level in the nucleus.

**Figure 5:**
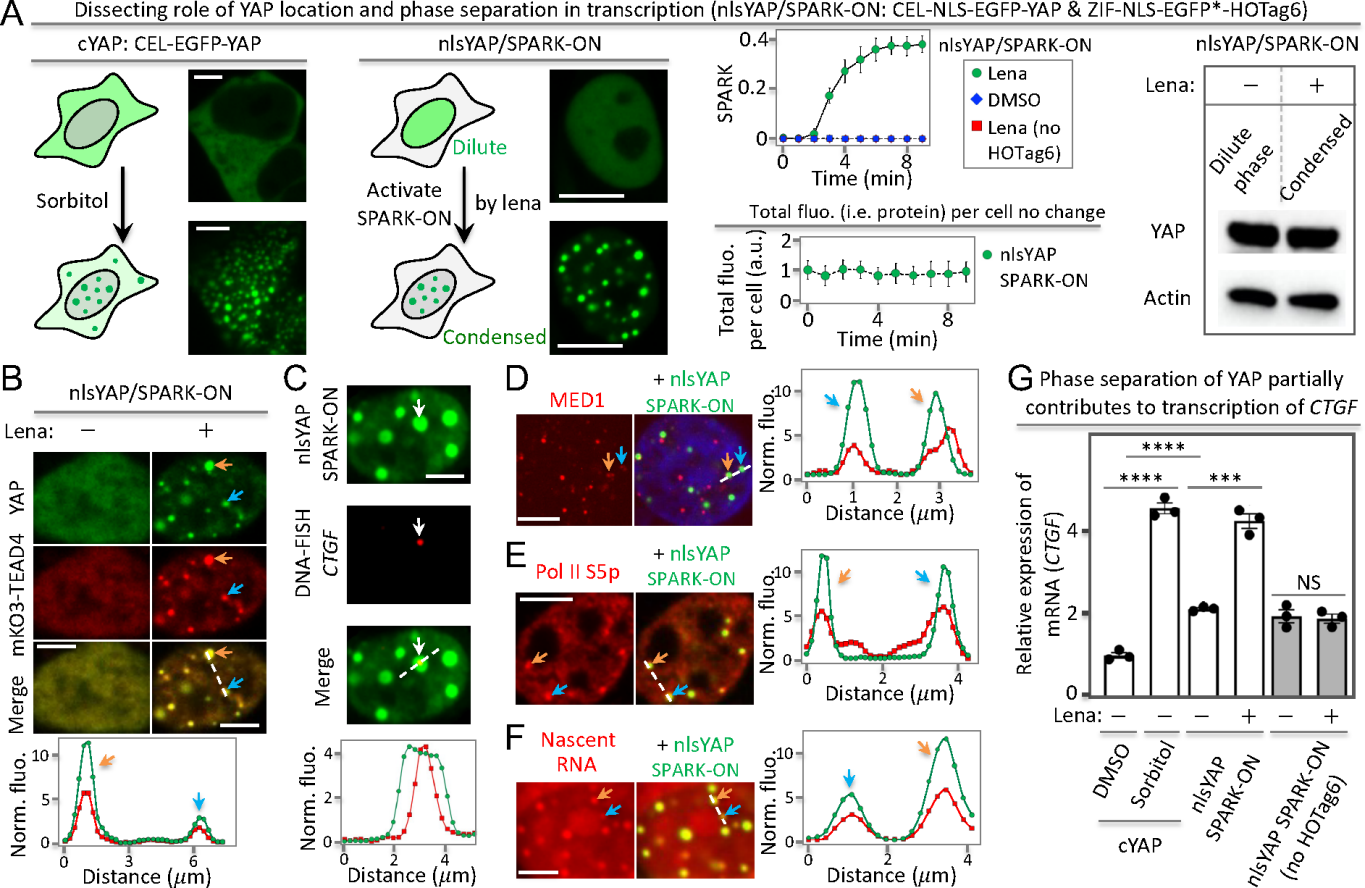
YAP phase separation promotes gene transcription. (A) YAP condensate formation via osmotic stress (sorbitol) or SPARK-ON-driven phase separation. nlsYAP: nuclear localized YAP (YAP fused to a nuclear localization signal peptide). (B–F). SPARK-ON-driven nlsYAP condensates are transcriptionally active, containing the DNA-binding partner TEAD (B), genomic DNA of its target gene *CTGF* (C), transcriptional machinery including the mediator (D) and Pol II (E), nascent RNA (F). (G) RT-qPCR analysis of YAP-target gene *CTGF* upon osmotic stress-induced vs SPARK-ON-induced phase separation of YAP. Data are the mean ± SD (*n* = 3). ****P < 0.0001. ***P < 0.001. NS, not significant. Scale bars: 5 μm (A–F).

First, we demonstrated that lenalidomide activated SPARK-ON and induced condensate formation within 3 to 4 min (Figure 5A). And the total fluorescence of nlsYAP/SPARK-ON underwent little change during phase separation, suggesting that the nlsYAP expression level was constant in the nucleus. Furthermore, Western blot analysis showed little difference in the YAP protein level between the dilute phase and the condensed phase. These data indicate that nlsYAP/SPARK-ON can drive YAP phase separation in the nucleus without changing the protein level. Our control experiments showed that DMSO did not induce nlsYAP phase separation. And lenalidomide alone could not drive nlsYAP phase separation using the nlsYAP/SPARK-ON control (no HOTag6). As a positive control, we also labeled YAP with CEL (cYAP), which was primarily localized on the cytoplasm as expected and formed condensates upon addition of sorbitol, consistent with previous studies (Figure 5A).

Next, we determined that the nlsYAP/SPARK-ON condensates contained the DNA-binding partner TEAD4 (Figure 5B) as well as genomic DNA of the YAP target gene *CTGF* using DNA FISH (Figure 5C, Supporting Excel File 1). These nlsYAP condensates also contained transcriptional machinery including MED1 and Pol II S5p (Figure 5D,E) and nascent RNA (Figure 5F). These data indicate that the nlsYAP/SPARK-ON condensates are transcriptionally active.

Finally, we determined the role of nlsYAP phase separation by RT-qPCR analysis of the mRNA level of *CTGF*. Lenalidomide-activated SPARK-ON-induced phase separation of nlsYAP enhanced the *CTGF* mRNA level by ∼2-fold (Figure 5G), whereas lenalidomide alone did not change the mRNA level. This indicates that phase separation, without a change in the YAP protein level in the nucleus, enhances the transcription of *CTGF*. Furthermore, we also compared the mRNA level of *CTGF* for nlsYAP in the dilute phase with cYAP that was localized in the cytoplasm, which showed that the former increased the *CTGF* mRNA level by ∼2-fold. This suggests that nuclear localization also enhances the transcription of *CTGF*. As a positive control, sorbitol also increased the *CTGF* mRNA level. Taken together, our data suggest that both nuclear translocation and phase separation enhance the transcription of *CTGF*, indicating that phase separation partially contributes to the transcription of the YAP target gene.

### SPARK-ON Enables the Formation of Transcriptionally Active TAZ Condensates

**Figure 6:**
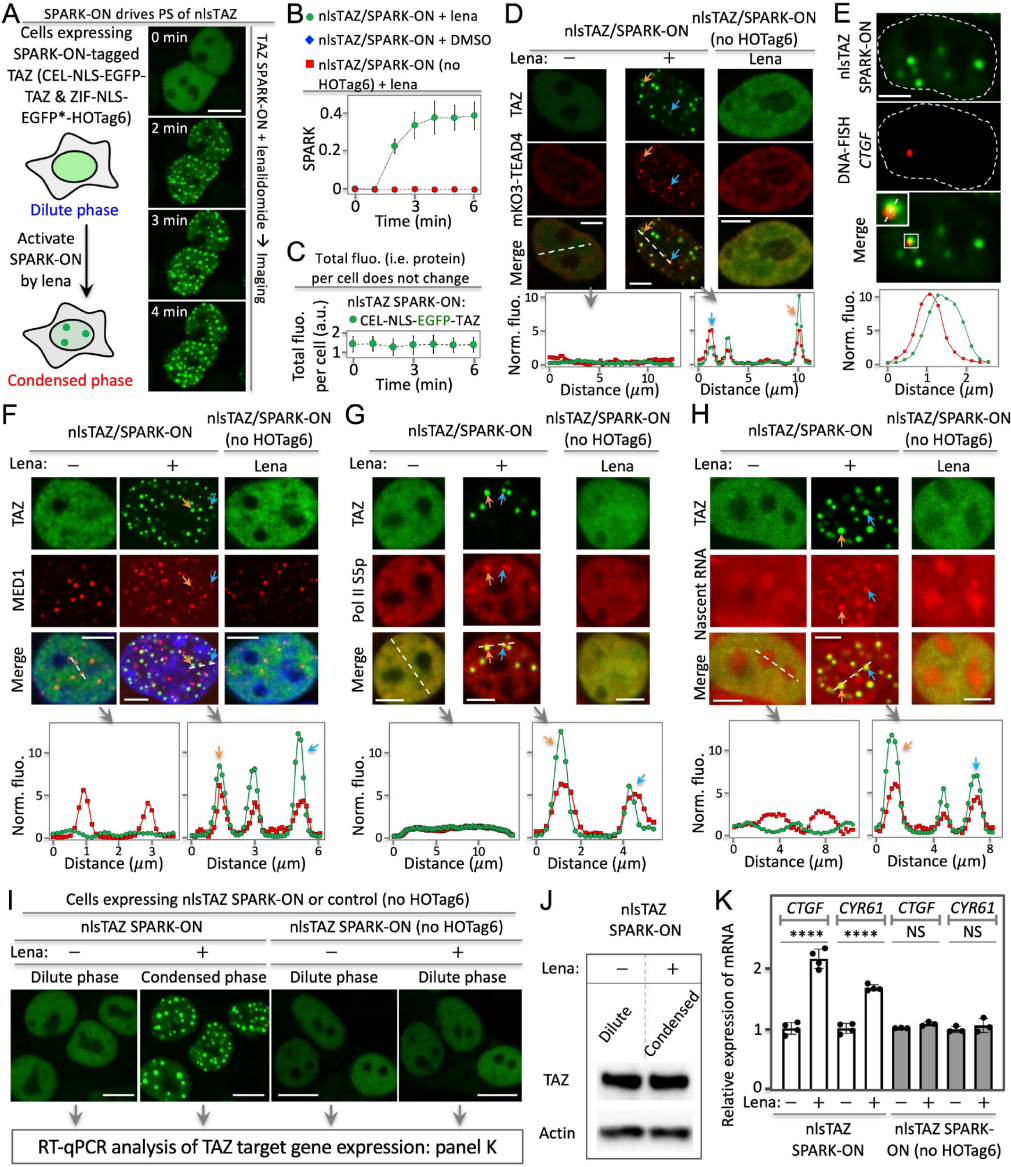
SPARK-ON drives TAZ phase separation that promotes transcription of the target genes. (A) Lenalidomide-activatable SPARK-ON induces nuclear TAZ (nlsTAZ) phase separation and condensate formation. (B) Quantitative analysis of SPARK-ON-driven nlsTAZ condensate formation over time, along with controls. (C) Total fluorescence of nlsTAZ per cell over time upon SPARK-ON. (D – H). SPARK-ON-driven nlsTAZ condensates are transcriptionally active, containing the DNA-binding partner TEAD (D), genomic DNA of its target gene *CTGF* (E), transcriptional machinery including the mediator (F) and Pol II (G), and nascent RNA (H). (I) Fluorescence images of SPARK-ON-driven nlsTAZ condensate formation. (J) Western blot showing the same protein level of TAZ upon SPARK-ON that is activated by lenalidomide. (K) RT-qPCR analysis of TAZ-target genes *CTGF* and *CYR61* upon SPARK-ON-induced phase separation of TAZ. Data are mean ± SD (*n* = 3). ****P < 0.0001. NS, not significant. Scale basr: 10 μm (A, I) and 5 μm (D–H).

To further demonstrate the SPARK-ON technology, we applied it to control TAZ condensate formation in living cells. Here we interrogated role of phase separation of TAZ on gene transcription. We applied SPARK-ON to manipulate a nuclear-localized TAZ (nlsTAZ/SPARK-ON) so that upon phase separation the protein abundance level of TAZ in the nucleus would not change. This will enable us to decouple the role of phase separation from the change in protein abundance in the nucleus. First, we demonstrated that SPARK-ON was able to drive the phase separation and condensate formation of TAZ in the nucleus. Lenalidomide activated SPARK-ON that drove TAZ phase separation, forming condensates in the nucleus within 2 min (Figure 6A,B). As a control, DMSO did not induce TAZ phase separation (Figure 6B). Furthermore, lenalidomide alone could not drive TAZ condensate formation, using the nlsTAZ/SPARK-ON control (no HOTag6) (Figure 6B). We also verified that the total fluorescence of TAZ did not change during phase separation (Figure 6C), which confirmed that the overall protein abundance level of nuclear TAZ was maintained at the same level before and after condensate formation.

Next, we determined that the nlsTAZ/SPARK-ON condensates contained the DNA-binding partner TEAD. Here, live-cell fluorescence imaging revealed that the SPARK-ON-induced TAZ condensates colocalized with the condensed TEAD4 structures (Figure 6D). Before lenalidomide activation of SPARK-ON, TAZ was evenly distributed in the nucleus, indicating that it was in the dilute phase. And TEAD4 was also evenly distributed without forming condensed structures. These data suggest that TAZ phase separation recruits TEAD4 into the condensates. As a control, lenalidomide alone did not induce TAZ phase separation using the TAZ/SPARK-ON control. And TEAD4 did not condense either.

We further determined that the nlsTAZ/SPARK-ON condensates contained genomic DNA of the TAZ target gene *CTGF*. We labeled the genomic DNA of *CTGF* using DNA FISH (fluorescence in situ hybridization).^50^ Confocal fluorescence imaging revealed that the TAZ condensates were associated with the genomic DNA of *CTGF* (Figure 6E, Supporting Excel File 1). These data suggest that the TAZ condensates bind genomic DNA, consistent with the above results that these condensates contain the DNA-binding partner TEAD (Figure 6D).

The nlsTAZ/SPARK-ON condensates contain transcriptional machinery and nascent RNAs. First, they contained MED1 (mediator of RNA polymerase II transcription subunit 1) based on immunofluorescence imaging (Figure 6F). Some TAZ/SPARK-ON condensates did not merge well with MED1, likely due to several factors that were discussed above for the YAP/SPARK-ON condensates.

Second, these condensates also contained RNA polymerase II (Pol II) S5p (Figure 6G). Finally, we determined that the nlsTAZ/SPARK-ON condensates contained nascent RNAs (Figure 6H). Here, we labeled nascent RNAs by incubating cells with uridine analog 5-ethynyluridine (EU) for 1 h so that EU is incorporated into newly transcribed RNA. Fluorescence imaging in the red channel revealed several punctate structures (Figure 6G). The small and round structures of nascent RNAs were colocalized with the TAZ condensates, suggesting that these TAZ condensates contain nascent RNAs.

Taken together, these data indicate that the SPARK-ON-induced TAZ condensates are transcriptionally active, containing the DNA-binding protein TEAD, genomic DNA, transcriptional machiner, and nascent RNA.

### Phase Separation of TAZ Promotes the Transcription of Its Core Target Genes

The above demonstration that the SPARK-ON-induced TAZ condensates are transcriptionally active paved the way for us to investigate the role of phase separation. We first prepared cells expressing nlsTAZ/SPARK-ON and treated them with or without lenalidomide, which showed the condensed or dilute phase of TAZ, respectively (Figure 6I). And Western blot analysis confirmed that the expression levels of TAZ had little effect on the dilute and the condensed phase (Figure 6J). RT-qPCR analysis of these samples revealed that the mRNA levels of *CTGF* and *CYR61*, two core target genes of TAZ, were significantly higher for the condensed TAZ than for the dilute TAZ (Figure 6K). These results suggest that TAZ phase separation increases the transcription of both *CTGF* and *CYR61*. We further showed that the lenalidomide-activatable SPARK-ON was able to quantitatively control TAZ activity in regulating the target gene *CTGF* expression by applying different levels of lenalidomide (Supporting Figure S16). As a control, we also prepared cells expressing the nlsTAZ/SPARK-ON control (no HOTag6), which did not form condensates upon addition of lenalidomide. And lenalidomide alone did not increase the transcription of the two target genes.

Therefore, our results suggest that TAZ phase separation, which can be regarded as a spatial reorganization of the transcription factor, enhances the transcription of its target genes. Our work demonstrates that this lenalidomide-activatable SPARK-ON enables us to decouple the role of phase separation in gene transcription from the change in the protein level, which will be well suited for interrogating the role of phase separation for many transcription factors.

## Discussion

The condensate biology has been an exciting area in cell and molecular biology over the past decade because it sheds light on a new structural entity that may play important roles in various cellular processes.^1,5,8,9,51,52^ The early groundbreaking work establishes that PS drives the formation of liquid-like condensates^2^ and that multivalent interactions drive PS.^3^ Currently, there are two basic biological questions regarding biomolecular condensates. First, do they have biological activities? Increasing evidence suggests that many biocondensates are biologically active in cell signaling.^1,5−9^ For example, the YAP and TAZ condensates are transcriptionally active and contain transcriptional machinery.^19,20^

The second key question for the condensate biology field is whether phase separation confers new or additional activities or is it simply a consequence of the protein level increase or molecular changes such as post-translational modifications? This question remains mostly unanswered due to conceptual and technical challenges.^21^ Many biomolecules, including transcription factors, form condensates via PS when protein levels reach above the saturation concentration. Biomolecular condensate formation can thus be divided into two steps: (1) the protein level increase above the saturation concentration and (2) phase separation via weak and multivalent interactions through IDRs. During phase separation, the biomolecules change from a diffuse state to a phase-separated state. The role of phase separation should thus be determined by comparing biological activities between these two states. An increase in the protein level such as a transcription factor is known to affect transcription. Therefore, to understand the role of phase separation, it is critical to decouple phase separation from the change in protein levels.

Previously, mutagenesis-based approaches have been used to block PS in order to determine the role of phase separation.^13,18^ However, this approach is often problematic because the IDRs that mediate PS are often within the activation domains that are essential to transcription. For example, the activation domain of GCN4 is known to interact with the mediator, and thus the mutations that are introduced into the AD of GCN4 may possibly affect the interaction with the transcriptional machinery.^58^ Another example is the work on YAP phase separation. One conclusion from this work is that the phase separation of YAP played key roles in the transcriptional regulation of YAP target genes.^18^ Unfortunately, the approach used in that work is problematic because in order to block PS the AD was deleted, while blocked PS would also largely reduce the transcriptional activity of YAP because AD is essential to gene transcription and thus its deletion would also affect the diffuse state of YAP.

To address the above challenge and decouple PS from protein-level changes without introducing mutations, we have developed chemogenetic tool SPARK-ON, which decouples phase separation from protein abundance. We first applied the structure-based approach and engineered a new chemogenetic heterodimer that is inducible by FDA-approved drugs collectively known as IMiDs. This new tool is complementary to and has several advantages over the FKBP-Frb pair-based systems in manipulating PS, including a smaller size (with one component as small as 31-aa, compared to ∼110-aa for FKBP and Frb) and a weaker interaction that better mimics weak interaction in most biomolecular condensates. By incorporating the multivalent tags from the *de novo*-designed coiled coils, we engineered the SPARK-ON tool that is appropriate for manipulating protein phase separation because first, the operating physical principle of SPARK-ON is based on multivalent PPIs that have been shown to drive PS,^3^ second, the SPARK-ON-induced condensates are highly dynamic and possess liquid-like properties, and third, the SPARK-ON tag exerts no or little perturbation on the function of transcription factors including YAP and TAZ. For example, our data show that SPARK-ON-tagged YAP and TAZ are both transcriptionally active and regulate the transcription of their core target gene *CTGF*. Furthermore, SPARK-ON-based manipulation of TAZ phase separation reveals the role of PS in promoting the transcription of *CTGF*, which is consistent with a previous study.^20^ On the other hand, our work clearly showed that PS of YAP promotes the transcription of the YAP target genes, reaching a similar conclusion with the previous work,^18^ though the previous work was based on the problematic approach as discussed above. Therefore, using SPARK-ON chemogenetic tools, we have obtained important biological findings on the role of phase separation of transcription factors on regulating gene expression.

We also demonstrated that SPARK-ON can be used to manipulate proteins other than TFs. We applied SPARK-ON to manipulate stress granule proteins including G3BP1, FUS, and TIA1. We combined our lenalidomide-inducible chemogenetic tool with the rapamycin-inducible system in manipulating protein phase separation because the two orthogonal small molecule-inducible heterodimers are compatible and can be combined to control protein interaction and phase separation. In particular, we used lenalidomide-inducible SPARK-ON and the rapamycin-inducible PPIs to interrogate the scaffold–client model of SG assembly. Our data indicate that the phase separation of SG scaffold protein G3BP1 recruits its clients including FUS and TIA1, which is consistent with the previous results using optogenetics-based approaches in manipulating G3BP1.^53^ Our SPARK-ON-based approach further reveals that phase separation of the client proteins including FUS and TIA1 does not recruit G3BP1. Therefore, our results, together with others, support the scaffold–client model of condensate assembly in which the phase separation of a scaffold recruits clients but not vice versa.^1,34^ Additionally, it has been reported that the optogenetic tools are not successful in manipulating full-length FUS and TIA1, though they were able to drive the condensate formation of truncated mutants containing LCD or IDR.^53,54^ The manipulation of full-length FUS and TIA1 phase separation is preferred since the LCD is not the entire protein after all.^55^ One advantage of using the chemogenetic systems is that it allows us to combine two chemogenetic systems so that we do not need to fuse the multivalent tag (HOTag) to proteins of interest (e.g., G3BP1), which maintains the stoichiometry of the protein before the induced phase separation.

In summary, the engineered chemogenetic SPARK-ON complements and offers several advantages to other approaches, including optogenetics and mutagenesis, in dissecting the role of phase separation in biological functions, which is one of the key basic questions in condensate biology. SPARK-ON can be applied to cytosolic proteins and transcription factors. Our work supports the scaffold–client model of SG assembly and reveals that the phase separation of YAP/TAZ promotes gene transcription. Therefore, the SPARK-ON chemogenetic tool is a versatile tool for the condensate biology field in dissecting the condensate assembly mechanisms and functional roles in cells.

## Supporting Information Available

Methods and materials; supplementary table S1: list of all constructs (PDF)
Movie S1. Lenalidomide-induced translocation of SOScat from cytosol to the plasma membrane (MP4)
Movie S2. Activation of ERK upon lenalidomide-induced translocation of SOScat (MP4)
Movie S3. ERK was not activated by DMSO (MP4)
Movie S4. ERK was not activated by lenalidomide itself without ZIF (MP4)
Movie S5. Time-lapse imaging of the zoom-in area corresponding to Figure 2B (MP4)
Movie S6. Time-lapse imaging showing the fusion and coalescence of protein droplets (MP4)
Movie S7. Time-lapse imaging showing the disassembly of protein droplets upon removal of lenalidomide (MP4)
Movie S8. Time-lapse imaging showing the phase separation of G3BP1 upon addition of rapamycin and the subsequent recruitment of FUS (MP4)
DNA-FISH probe information (XLSX)
