# Supplementary material for: Chemogenetic Minitool for Dissecting the Roles of Protein Phase Separation
Source: ACS Cent Sci. 2023 Jul 7;9(7):1466–79. doi: 10.1021/acscentsci.3c00251 (PMC10375881; doi:10.1021/acscentsci.3c00251)
Supplement: Supplementary file 1 — oc3c00251_si_002.pdf [file oc3c00251_si_002.pdf]

A chemogenetic mini-tool for dissecting roles of protein phase separation

Chan-I Chung[1, 2], Junjiao Yang[1, 2], Xiaokun Shu[1, 2, *]

[1]Department of Pharmaceutical Chemistry, University of California – San Francisco, San Francisco, California, 94158, USA.

[2]Cardiovascular Research Institute, University of California – San Francisco, San Francisco, California, 94158, USA

[*]Correspondence to: Xiaokun Shu PhD (email: xiaokun.shu@ucsf.edu)

Supplementary Materials

Materials and Methods

Plasmid construction. CRBN, IKZF1, G3BP1, FUS, TIA1, SOS2, TEAD4, NPM1 were amplified from The CCSB Human ORFeome8.1 Collection (donated by Marc Vidal). YAP and TAZ was amplified from HEK293 cDNA library. pENTR-ERK-KTRClover was a gift from Markus Covert (Addgene plasmid # 59138). pcDNA3-FLAG-DDB1 was a gift from Yue Xiong (Addgene plasmid # 19918). mKO2-N1 was a gift from Michael Davidson & Atsushi Miyawaki (Addgene plasmid # 54625). mKO3 was obtained by introducing the M176F mutation to mKO2 for brighter fluorescence in cells[1,2]. pHR_SFFV was a gift from Wendell Lim (Addgene plasmid # 79121). The details of all constructs in this study are listed in Table S1. All plasmid constructs were created by standard restriction enzyme digestion and ligation method and confirmed by exhaustively sequencing the cloned fragments. CRBN and its C-terminal domain were fused with 3x Flag tag cloned into pcDNA3.1 plasmid resulting in 3xFlag-CRBN and 3xFlag-CEL. SOS2 catalytic domain and membrane targeting motif, CAAX were fused to CEL and zinc finger domain 2 of IKZF1 resulting in CEL-IFP2-SOScat and ZIF-RFP-CAAX. To create CEL-EGFP-HOTag3 fusions, the C-terminal domain of CRBN protein was amplified and cloned into pcDNA3 containing EGFP-HOTag3 resulting in CEL-EGFP-HOTag3. Similar procedures were carried out to produce ZIF-EGFP-HOTag6. To create the SparkDrop-Frb, a Frb domain was inserted into CEL-EGFP-HOTag3. IFP2-G3BP1 and FKBP- IFP2-G3BP1 were created by linking the DNA sequence of G3BP1 to IFP2, and G3BP1 to FKBP-IFP2. FUS-mKO3, FUS-mKO3-FKBP, mKO3-TIA1, FKBP-mKO3-TIA1, mKO3-TEAD4, NPM1-EGFP were created in a similar procedure. CEL-EGFP-YAP, CEL-NLS-EGFP-YAP and CEL-NLS-EGFP-TAZ were created by replacing HOTag3 in CEL-EGFP-HOTag3 or CEL-NLS-EGFP-HoTag3 with YAP and TAZ.

Cell culture and Live cell imaging. HEK293 cells were cultured in Dulbecco's Modified Eagle medium (DMEM) high glucose (Gibco 11965092), supplemented with 10% Fetal Bovine Serum (FBS) heat inactivated (Gibco 10438026), penicillin (100 units/mL) and streptomycin (100 μg/mL) (Gibco 15140122). HEK293 cells were transiently transfected with the plasmid using

calcium phosphate transfection reagent. Cells were grown in 35 mm glass bottom, four-compartments dishes (Greiner Bio-One 627870). Transfection was performed when cells were cultured to ~50% confluence. Cells were imaged 24 hours after transient transfection. Time-lapse imaging was performed with the aid of an environmental control unit incubation chamber (InVivo Scientific), which was maintained at 37 °C and 5% CO_2. Lenalidomide (TargetMol, T1642) were carefully added to the cells in the incubation chamber when the time-lapse imaging was started.

Confocal microscopy. Samples were imaged on a Nikon Eclipse Ti inverted microscope equipped with a Yokogawa CSU-W1 confocal scanner unit (Andor), a digital CMOS camera ORCA-Flash4.0 (Hamamatsu), a ASI MS-2000 XYZ automated stage (Applied Scientific Instrumentation) and Nikon Plan Apo λ 20X air (N.A. 0.75), Nikon Apo TIRF 60X oil (N.A. 1.49) and CFI Plan Apo λ 100X oil (N.A. 1.45) objectives. Laser inputs were provided by an Integrated Laser Engine (Spectral Applied Research) equipped with laser lines (Coherent) 405 nm for Hoechst imaging, 488 nm for GFP imaging, 561 nm for RFP imaging and 640 nm for IFP imaging. The confocal scanning unit was equipped with the following emission filters: 460/50 nm for Hoechst imaging, 525/50-nm for GFP imaging, 610/60-nm for RFP imaging and 732/68 nm for IFP imaging. Image acquisition was controlled by the NIS-Elements Ar Microscope Imaging Software (Nikon).

Flag-Tag pull-down and immunoblotting. HEK293 cells expressing 3xFlag-CEL, 3xFlag-CRBN with or without DDB1 overexpression were lysed in TBS (50 mM Tris HCl, pH 7.4, with 150 mM NaCl, 1 mM EDTA, and 1% TRITON X-100) with protease inhibitor cocktail (Roche), and the cell lysates were incubated with anti-flag magnetic beads (Sigma, M8823) at 4 °C for 2 hrs. Proteins bound to magnetic beads were eluted with SDS sample buffer, then resolved using SDS-PAGE. The bound DDB-1 proteins were detected by anti-DDB1 antibody (Cell Signaling, 5428S).

Immunofluorescence and nascent-RNA labelling. HEK293 cells were plated on fibronectin (Sigma-Aldrich F2006) pre-coated 8-well chambered coverglass (Nunc Lab-Tek II, 155409) and were infected with YAP/SPARK-ON, nlsYAP/SPARK-ON and nlsTAZ/SPARK-ON plasmids. After treating cells with 1 μM lenalidomide for 2 hrs, the cells were fixed with 4 % paraformaldehyde in PBS, permeabilized with PBST (0.5% Triton X-100 in PBS) and blocked with 2% BSA and 10% goat serum in PBS. The cells were next incubated with anti-RNA polymerase II CTD repeat YSPTSPS (phospho S5) antibody (1:200 dilution, Abcam, ab5408) in blocking buffer at 4 °C for overnight, Med1 antibody (1:2000 dilution Santa Cruz sc-74475) at room temperature for 3 hrs. After washing three times with PBST, the cells incubated with Alexa Fluor 555-conjugated secondary antibodies (1:200 dilution, Abcam, ab150114) at room temperature for 1 hr. After washing with PBST three times, 1 μg/mL Hoechst 33342 was added to each well and the cells were imaged after 10 mins. The nascent RNA was labelled with Click-iT RNA Alexa Fluor 594 imaging kit (Thermo Fisher, C10330) following manufacturer's protocol. HEK293 cells expressing SPARK-ON, YAP/ SPARK-ON, nlsYAP/ SPARK-ON, nls-TAZ/SPARK-ON and EGFP-NPM1 were treated with 1 μM lenalidomide for 2 hrs, then labelled with 1 mM 5-ethynyl uridine for 1 hr. After fixed with 4 % paraformaldehyde in PBS, permeabilized with PBST (0.5% Triton X-100 in PBS), the cells were incubated with Click-iT reaction cocktail at room temperature for 30 mins to visualize 5-ethynyl uridine.

DNA-FISH combined with immunofluorescence. DNA FISH and Primer Exchange Reaction (PER) were performed as described[3]. *CTGF* FISH probes were designed with PaintSHOP[4].The probe sequence used in this study listed in Supplementary Excel File 1. PER mix contains 10mM MgSO4, 0.3 mM dNTP (A, C, T only), Clean G 0.1μM, Bst polymerase 6 units/μL, Hairpin 0.8 μM. After 37°C 15 mins incubation, add *CTGF* probe to final concentration 1 μM. After 37°C 1 hr incubation, heat inactivation at 80°C 20 mins. The *CTGF* probes after PRE were purified with DNA clean & concentrator kit (Zymo research, D4014) for further use.

HEK293T cells grown on fibronectin pre-coated 8-well chambered coverglass were transfected with nlsYAP/SPARK-ON and nlsTAZ/SPARK-ON plasmid. 2 hrs after challenged with 1 μM lenalidomide, cells were fixed in 4% (wt/vol) paraformaldehyde for 10 min at room temperature. After fixation, cells were permeabilized in 1xPBS with 0.5% Triton X-100 for 10 min at room temperature. Cells were incubated in 0.1 N HCl for 5 min and washed in 2× SSC with 0.1% Tween-20 for 2 min twice. After incubated in 2× SSCT with 50% formamide for 1 hr at 60 °C. Then the well were loaded with 150 µl of ISH solution (2× SSCT, 50% (vol/vol) formamide, 10% dextran sulfate, 400 ng/µl RNase A and 100 nM probe. After denaturation at 80 °C for 3 min, cells were incubated overnight at 44 °C. After hybridization, cells were washed in 2× SSC with 0.1% Tween-20 at 60°C for 4 times and rinsed in 1× PBS for 1 minute. The cells then incubated with 1uM Atto 565 labeled imager in PBS at 37°C for 1 hr and washed in 1× PBS twice. Then block the same samples with 2% BSA and 10% goat serum in PBS at room temperature for 30 mins. The cells were stained with anti-GFP antibody (1:200 dilution, Abcam, Ab290) for 3 hrs at room temperature. After washing three times with PBST, the cells were incubated with Alexa Fluor 488-conjugated secondary antibodies (1:200 dilution, Cell Signaling, #4412) at room temperature for 1 hr. The confocal images of 488 nm for nlsYAP/SPARK-ON and nlsTAZ/SPARK-ON, 561 nm for *CTGF* FISH were taken together.

Lentivirus preparation. YAP/SPARK-ON, YAP/SPARK-ON control (no nls-HOTag6), nlsYAP/SPARK-ON, nlsYAP/SPARK-ON control (no nls-HOTag6), nls-TAZ/SPARK-ON, nls-TAZ/SPARK-ON control (no nls-HOTag6) lentiviral plasmids were co-transfected with pAX2 and pVSVG at 3:2:1 ratio into HEK293T cells using polyethylenimine (MilliporeSigma 764965). Lentiviruses were harvested after 48 hours.

Western blot. HEK293T cells expressing nlsYAP/SPARK-ON and nlsTAZ/SPARK-ON were grown on 24 well plates, washed once with PBS, lysed with 100 µL lysis buffer (Cell signaling 9803S) at room temperature for 1 minute, mixed with 37.5 µL NuPAGE LDS sample buffer (NP0007) and 15 µL NuPAGE sample reducing agent (NP0009) and incubated at 70ºC for 10 minutes. Protein samples were resolved on NuPAGE 4-12% Bis-Tris gel (NP0336), then transferred to nitrocellulose membrane under 25 V for 45 min in a Bio-rad Trans-Blot Turbo machine. The membrane was then blocked in 5% skim milk at room temperature for 1 hour, incubated with a YAP rabbit polyclonal antibody (Cell signaling 4912S, 1000X) and TAZ rabbit polyclonal antibody (Sigma-Aldrich HPA007415, 1000X) at 4ºC over-night, washed 3 times in PBST (0.1% Tween-20 in PBS), each time for 5 minutes, incubated with an HRP-conjugated anti-rabbit secondary antibody (Cell signaling 7074S, 3000X) and washed 3 times, each time for 5 minutes. HRP chemiluminescent substrate (Thermo scientific 34580) was added to the membrane, incubated at room temperature for 5 minutes, then imaged using Bio-Rad Chemidoc XRS system or film (Prometheus 30-507L) exposure. For β-actin, the staining process was

similar, but the primary antibody (Santa Cruz sc-47778, 3000X) and HRP-conjugated anti-mouse secondary antibody (Cell signaling 7076S, 3000X) were incubated for 1 hour at room temperature.

RT-qPCR. HEK293 cells were infected with YAP/SPARK-ON, nlsYAP/SPARK-ON, nlsTAZ/SPARK-ON and their control (no nls-HOTag6) in presence of 10 µg/mL Polybrene (Sigma-Alderich, H9268). 1 days after infection, the cells with green fluorescence were collected by FACS sorting (BD FACSAria Fusion) and seed $1x10^5$ cells per cm^2. 2 days after FACS sorting, the cells were challenged with 1 µM lenalidomide, 0.01% DMSO or 200 mM D-Sorbitol (Sigma-Aldrich S1876) for 3 hrs. The total RNA was extracted using a Direct-zol RNA MicroPrep (Zymo Reserch, R2060) and converted to complimentary DNA using a SuperScript™ IV Reverse Transcriptase (Invitrogen, 18090010). The RT-qPCR was carried out on a CFX96 Touch Real-Time PCR Detection System using iTaq Universal SYBR Green Supermix (Bio-Rad, 1725121) with a *GAPDH* control. The following primers were used:

	Forward primer	Reverse primer
GAPDH	GTCTCCTCTGACTTCAACAGCG	CTCTTCCTCTTGTGCTCTTGCTG
CTGF	AGGAGTGGGTGTGTGACGA	CCAGGCAGTTGGCTCTAATC
CYR61	CCTCGGCTGGTCAAAGTTAC	TTTCTCGTCAACTCCACCTC

Image analysis. For analysis of the SPARK signal, images were processed in ImageJ. The sum of droplets pixel fluorescence intensity and the cells pixel intensity were scored using Analyze Particle function in imageJ. Quantitative data was plotted in EXCEL.

Reference:

(1) Mastop, M., Bindels, D. S., Shaner, N. C., Postma, M., Gadella, T. W. J., and Goedhart, J. Characterization of a spectrally diverse set of fluorescent proteins as FRET acceptors for mTurquoise2. *Sci. Rep.* 2017, *7*, 1–18.
(2) Tsutsui, H., Karasawa, S., Okamura, Y., and Miyawaki, A. Improving membrane voltage measurements using FRET with new fluorescent proteins. *Nat. Methods* 2008, *5*, 683–685.
(3) Kishi, J. Y., Lapan, S. W., Beliveau, B. J., West, E. R., Zhu, A., Sasaki, H. M., Saka, S. K., Wang, Y., Cepko, C. L., and Yin, P. SABER amplifies FISH: enhanced multiplexed imaging of RNA and DNA in cells and tissues. *Nat. Methods* 2019, *16*, 533–544.
(4) Hershberg, E. A., Camplisson, C. K., Close, J. L., Attar, S., Chern, R., Liu, Y., Akilesh, S., Nicovich, P. R., and Beliveau, B. J. PaintSHOP enables the interactive design of transcriptome- and genome-scale oligonucleotide FISH experiments. *Nat. Methods* 2021, *18*, 937–944.

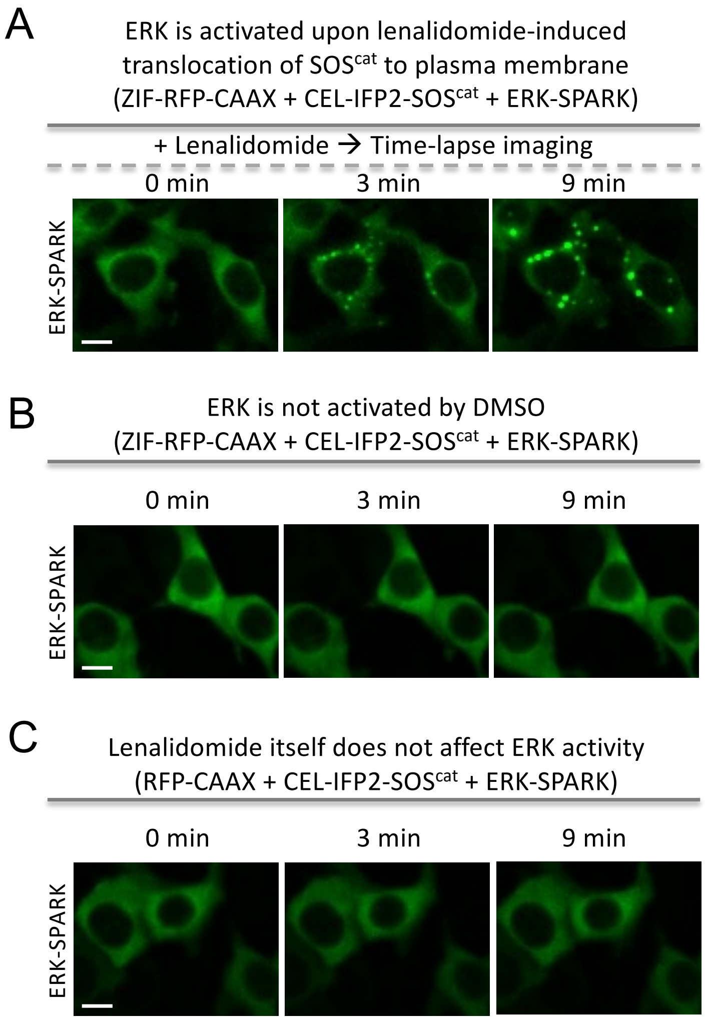

Fig. S1 Lenalidomide-inducible CEL and ZIF heterodimer induces SOS^{cat} translocation and activates ERK. (A) ERK is activated by lenalidomide-induced transloation of SOS^{cat} to the plasma membrane. ERK activity is visualized by a previously reported ERK activity sensor ERK-SPARK. Cells were transfected with ZIF-RFP-CAAX, CEL-IFP2-SOS^{cat}, and the ERK activity reporter ERK-SPARK, which is a GFP phase separation-based ERK activity reporter. Upon ERK activation, ERK-SPARK is phosphorylated and forms bright GFP droplets. (B) ERK is not activated by DMSO. Cells were transfected with ZIF-RFP-CAAX, CEL-IFP2-SOS^{cat}, and the ERK activity reporter ERK-SPARK. (C) Lenalidomide itself does not affect ERK activity. Cells were transfected with RFP-CAAX, CEL-IFP2-SOS^{cat}, and the ERK activity reporter ERK-SPARK. Scale bar: 10 μm.

HOTag3 (hexamer) GEIAKSLKEIAKSLKEIAWSLKEIAKSLKG
HOTag6 (tetramer) TLREIEELLRKIIEDSVRSVAELEDIEKWLKKI

Fig. S2 Protein sequence of HOTag3 and HOTag6.

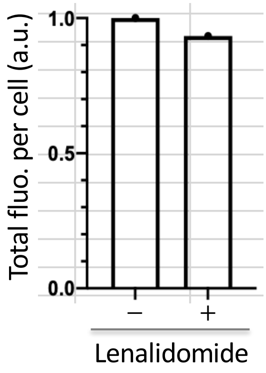

Fig. S3 Total protein level of SOScat did not increase and had little change upon addition of lenalidomide in cells expressing the constructs.

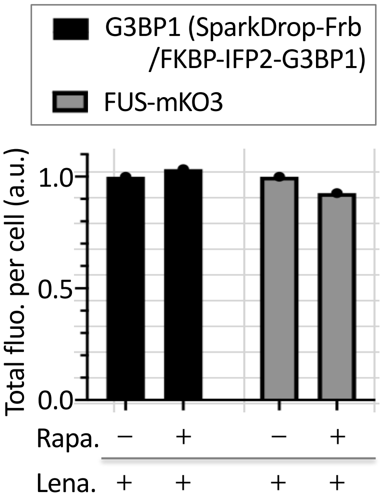

Fig. S4. Total protein levels of G3BP1 and FUS had little change when G3BP1 phase separation was induced in cells expressing the SPARK-ON constructs.

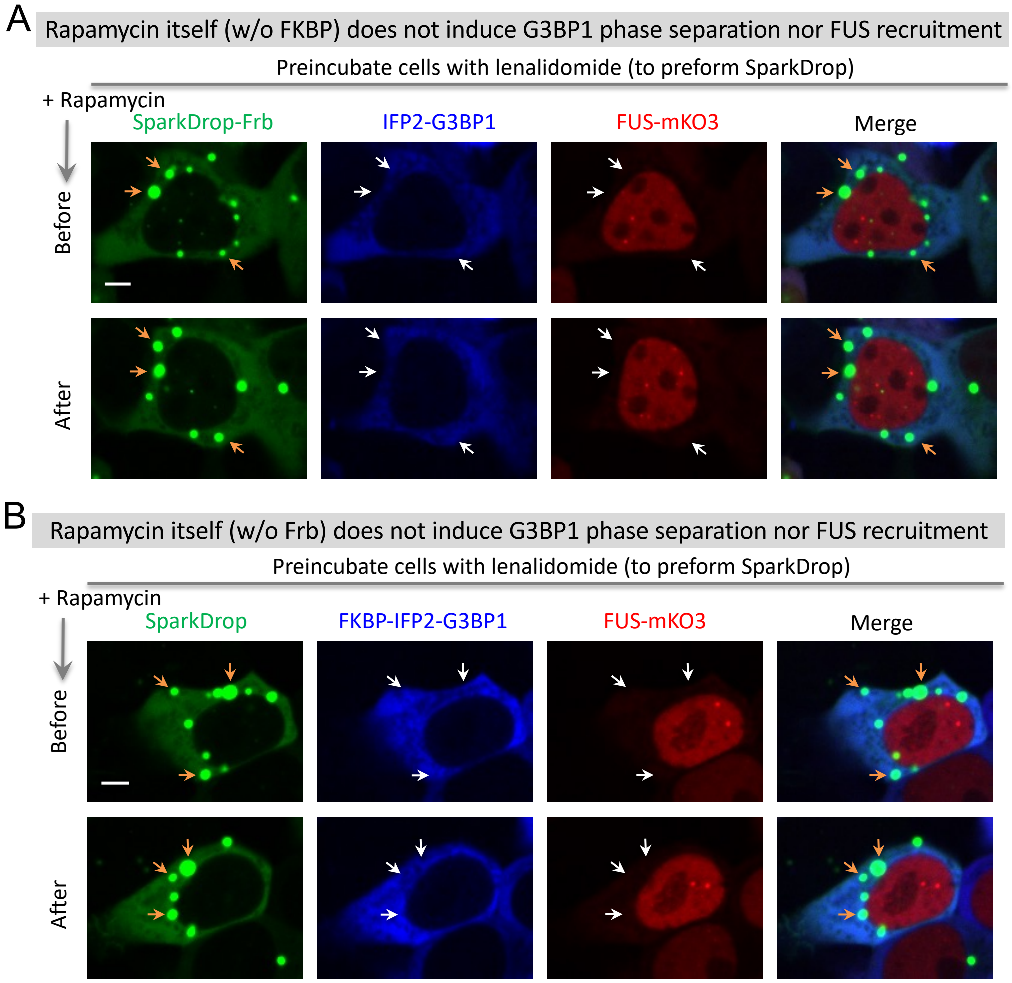

Fig. S5. Rapamycin itself without FKBP (A) or Frb (B) cannot induce G3BP1 phase separation nor FUS recruitment. (A) HEK293 cells were transfected with SparkDrop-Frb (i.e. CEL-Frb-EGFP-HOTag3, ZIF-EGFP-HOTag6), IFP2-G3BP1, and FUS-mKO3. The cells were preincubated with lenalidomide to form droplets. Then rapamycin was added to the cells. (B) HEK293 cells were transfected with SparkDrop (i.e. CEL-EGFP-HOTag3, ZIF-EGFP-HOTag6), FKBP-IFP2-G3BP1, and FUS-mKO3. The cells were preincubated with lenalidomide to form droplets. Then rapamycin was added to the cells. Scale bar: 5 μm.

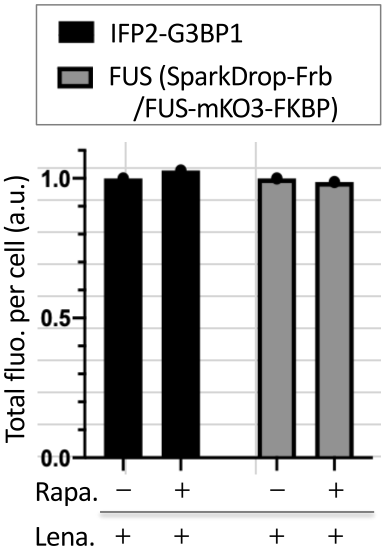

Fig. S6. Total protein levels of G3BP1 and FUS had little change when FUS phase separation was induced in cells expressing the SPARK-ON constructs.

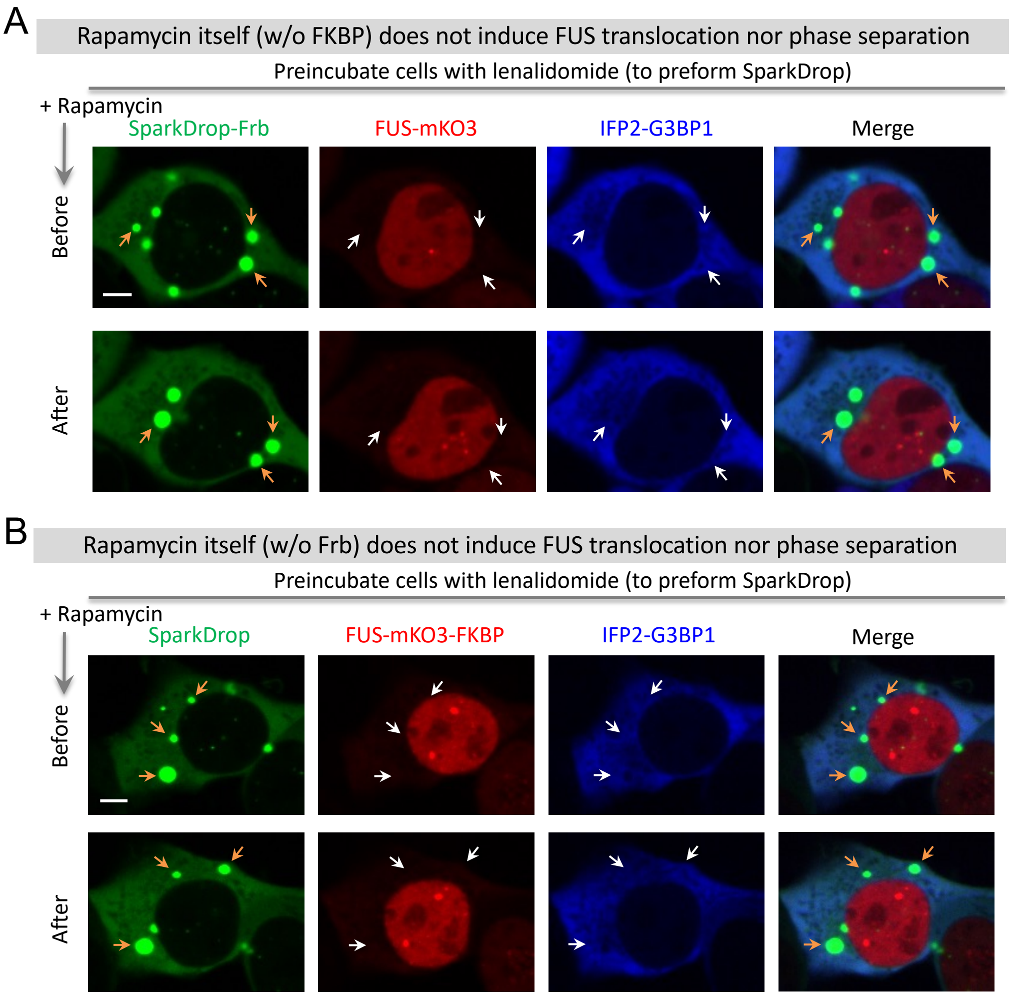

Fig. S7 Rapamycin itself without FKBP (A) or Frb (B) cannot induce FUS phase separation nor G3BP1 recruitment. (A) HEK293 cells were transfected with SparkDrop-Frb (i.e. CEL-Frb-EGFP-HOTag3, ZIF-EGFP-HOTag6), FUS-mKO3, and IFP2-G3BP1. The cells were preincubated with lenalidomide to form droplets. Then rapamycin was added to the cells. (B) HEK293 cells were transfected with SparkDrop (i.e. CEL-EGFP-HOTag3, ZIF-EGFP-HOTag6), FUS-mKO3-FKBP, and IFP2-G3BP1. The cells were preincubated with lenalidomide to form droplets. Then rapamycin was added to the cells. Scale bar: 5 μm.

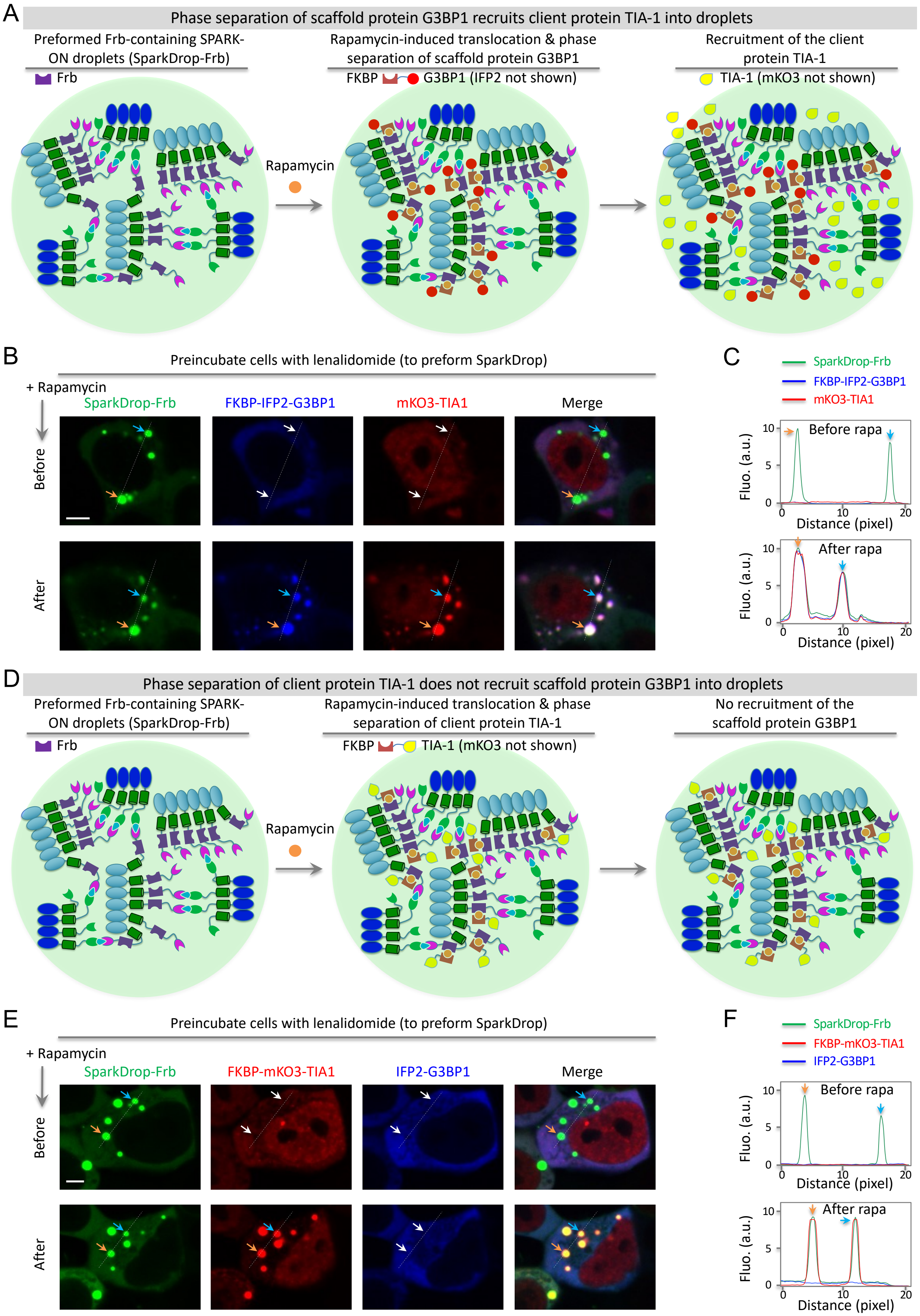
A
Phase separation of scaffold protein G3BP1 recruits client protein TIA-1 into droplets
Preformed Frb-containing SPARK-ON droplets (SparkDrop-Frb)
Frb
Rapamycin-induced translocation & phase separation of scaffold protein G3BP1
FKBP
G3BP1 (IFP2 not shown)
Recruitment of the client protein TIA-1
TIA-1 (mKO3 not shown)
Rapamycin
B
Preincubate cells with lenalidomide (to preform SparkDrop)
+ Rapamycin
SparkDrop-Frb
FKBP-IFP2-G3BP1
mKO3-TIA1
Merge
Before
After
C
SparkDrop-Frb
FKBP-IFP2-G3BP1
mKO3-TIA1
Before rapa
After rapa
Fluo. (a.u.)
Distance (pixel)
D
Phase separation of client protein TIA-1 does not recruit scaffold protein G3BP1 into droplets
Preformed Frb-containing SPARK-ON droplets (SparkDrop-Frb)
Frb
Rapamycin-induced translocation & phase separation of client protein TIA-1
FKBP
TIA-1 (mKO3 not shown)
No recruitment of the scaffold protein G3BP1
Rapamycin
E
Preincubate cells with lenalidomide (to preform SparkDrop)
+ Rapamycin
SparkDrop-Frb
FKBP-mKO3-TIA1
IFP2-G3BP1
Merge
Before
After
F
SparkDrop-Frb
FKBP-mKO3-TIA1
IFP2-G3BP1
Before rapa
After rapa
Fluo. (a.u.)
Distance (pixel)

Fig. S8. **Phase separation of SG scaffold protein G3BP1 can recruit client proteins but not vice versa.**

(**A** – **C**). SparkDrop-induced (no stress stimuli such as arsenite) phase separation of G3BP1 recruits FUS. A, schematic of the experimental design with observed results. B, time-lapse fluorescence images before and after (30 min) addition of 30 nM rapamycin to HEK293 cells expressing SparkDrop-Frb (i.e. constructs of CEL-Frb-EGFP-HOTag3 and ZIF-EGFP-HOTag6), FKBP-IFP2-G3BP1 and FUS-mKO3. C, fluorescence intensity plot against distance (dashed lines in panel B). The cells were preincubated with 1 μM lenalidomide for 30 minutes.

(**D** – **F**). SparkDrop-induced (no stress stimuli such as arsenite) phase separation of FUS does not recruit G3BP1. D, schematic of the experimental design with observed results. E, time-lapse fluorescence images before and after (30 min) addition of 30 nM rapamycin to HEK293 cells expressing SparkDrop-Frb (i.e. constructs of CEL-Frb-EGFP-HOTag3 and ZIF-EGFP-HOTag6), FUS-mKO3-FKBP and IFP2-G3BP1. F, fluorescence intensity plot against distance (dashed lines in panel E). The cells were preincubated with 1 μM lenalidomide for 30 minutes. Scale bar: B and E, 5 μm.

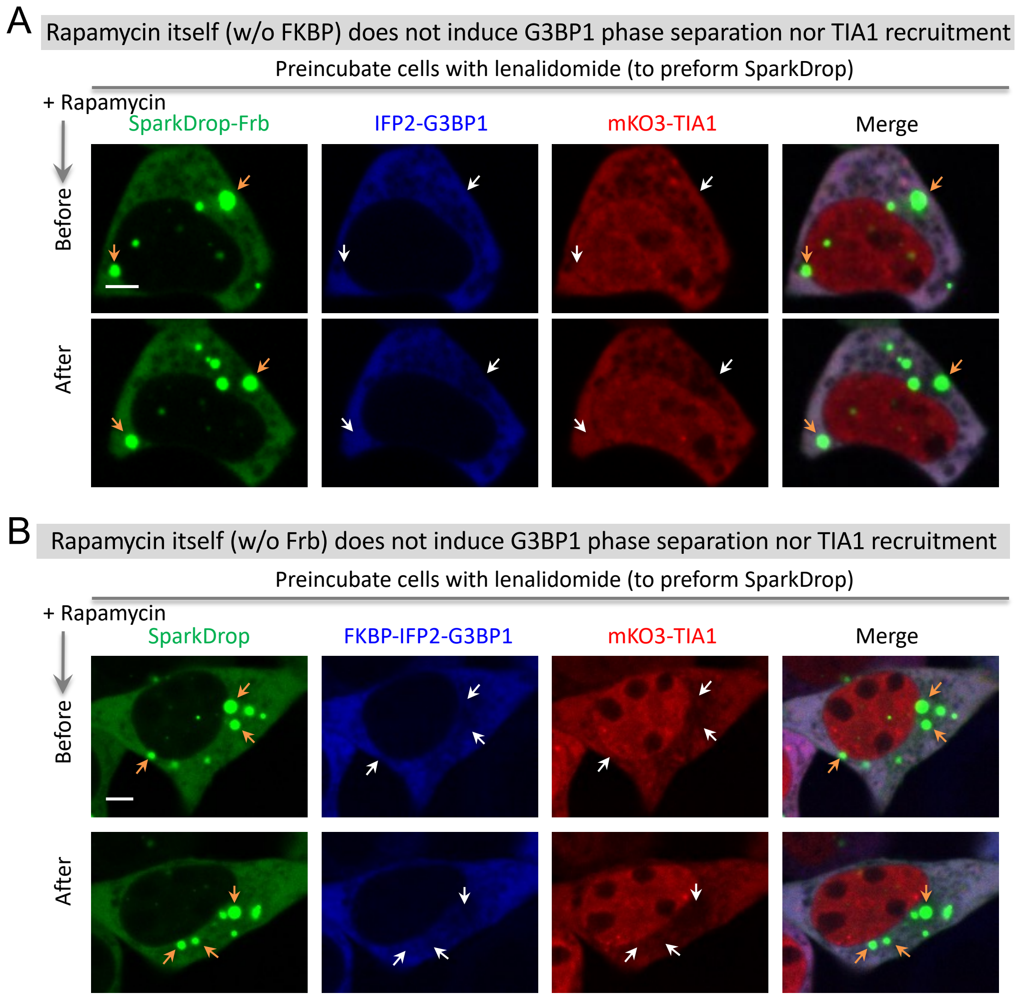

Fig. S9. Rapamycin itself without FKBP (A) or Frb (B) cannot induce G3BP1 phase separation nor TIA-1 recruitment. (A) HEK293 cells were transfected with SparkDrop-Frb (i.e. CEL-Frb-EGFP-HOTag3, ZIF-EGFP-HOTag6), IFP2-G3BP1, and mKO3-TIA1. The cells were preincubated with lenalidomide to form droplets. Then rapamycin was added to the cells. (B) HEK293 cells were transfected with SparkDrop (i.e. CEL-EGFP-HOTag3, ZIF-EGFP-HOTag6), FKBP-IFP2-G3BP1, and mKO3-TIA1. The cells were preincubated with lenalidomide to form droplets. Then rapamycin was added to the cells. Scale bar: 5 µm.

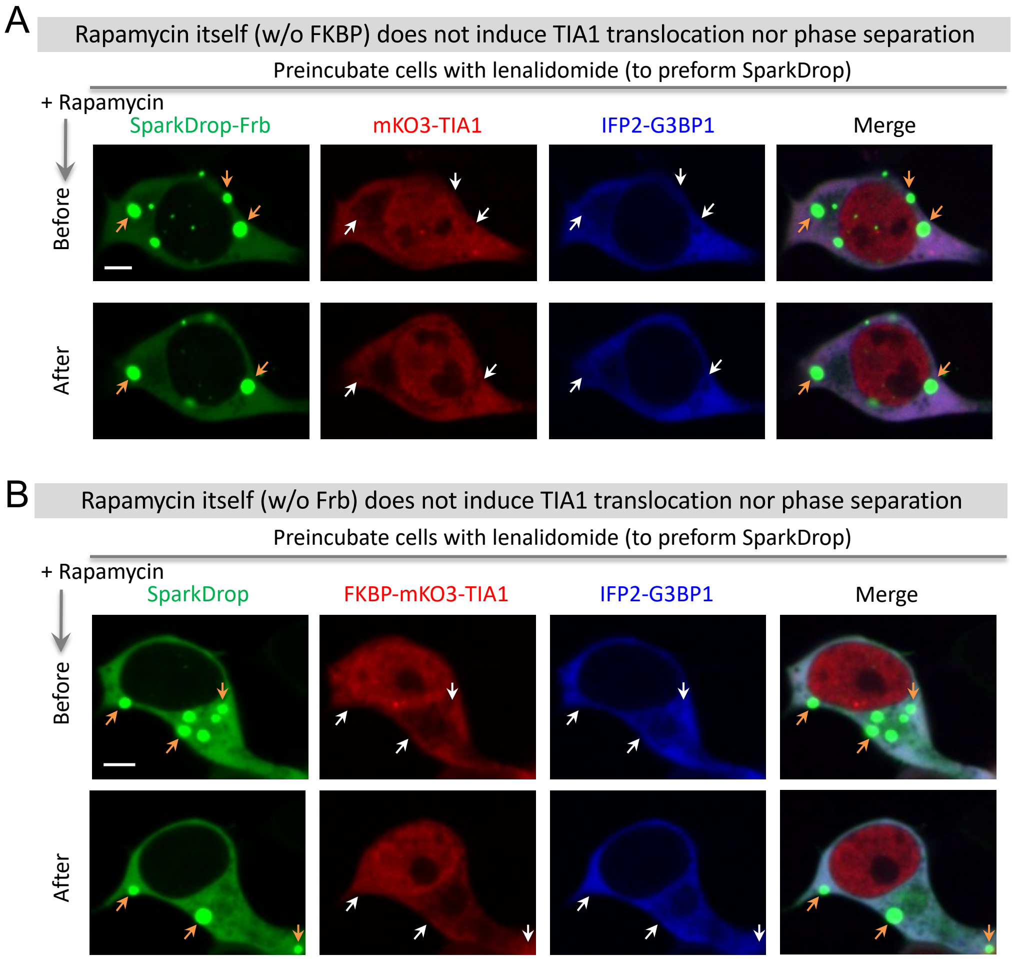

Fig. S10. Rapamycin itself without FKBP (A) or Frb (B) cannot induce TIA1 phase separation nor G3BP1 recruitment. (A) HEK293 cells were transfected with SparkDrop-Frb (i.e. CEL-Frb-EGFP-HOTag3, ZIF-EGFP-HOTag6), mKO3-TIA1, and IFP2-G3BP1. The cells were preincubated with lenalidomide to form droplets. Then rapamycin was added to the cells. (B) HEK293 cells were transfected with SparkDrop (i.e. CEL-EGFP-HOTag3, ZIF-EGFP-HOTag6), FKBP-mKO3-TIA1, and IFP2-G3BP1. The cells were preincubated with lenalidomide to form droplets. Then rapamycin was added to the cells. Scale bar: 5 µm.

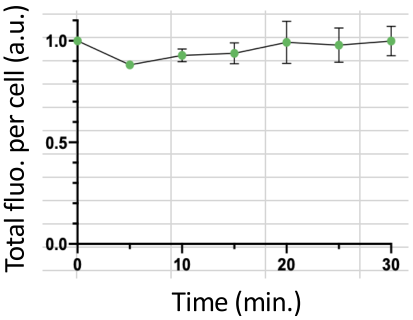

Fig. S11. Total protein levels of YAP did not increase and had little change over the course of SPARK-ON-induced YAP phase separation in cells expressing the YAP/SPARK-ON constructs. Data are mean ± SD (n = 3).

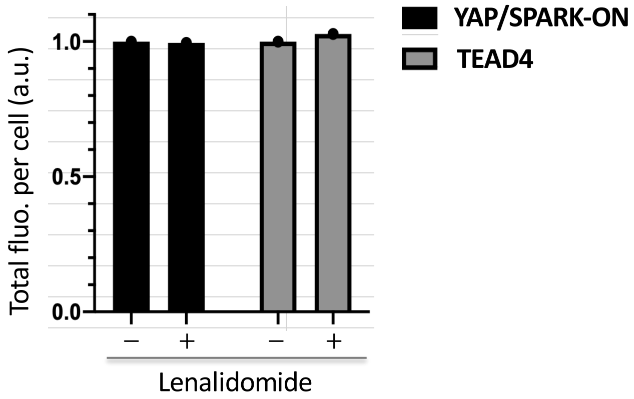

Fig. S12. Total protein levels of YAP and TEAD4 had little change when YAP phase separation was induced and TEAD4 was recruited to the YAP condensates in cells expressing the SPARK-ON constructs.

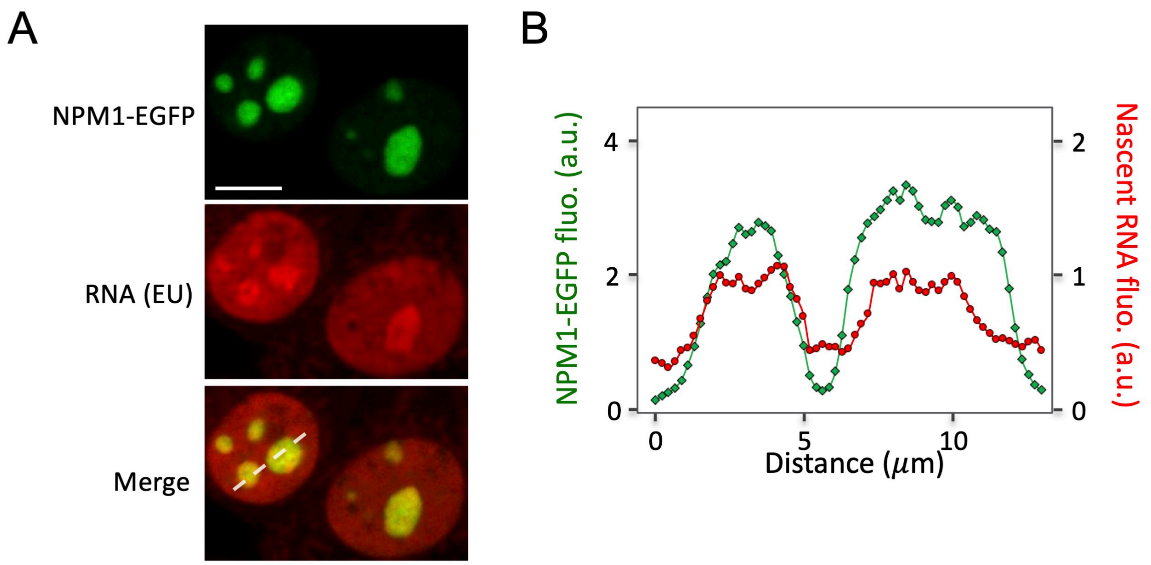

Fig. S13 Nascent RNAs in the nucleoli are labeled by 5'-EU. A, Fluorescence images of nucleoli and nascent RNA labeling. B, Fluorescence intensity of nucleoli (NPM1-mEGFP) and nascent RNA against position shown by dashed line in A. Scale bar, 5 μm.

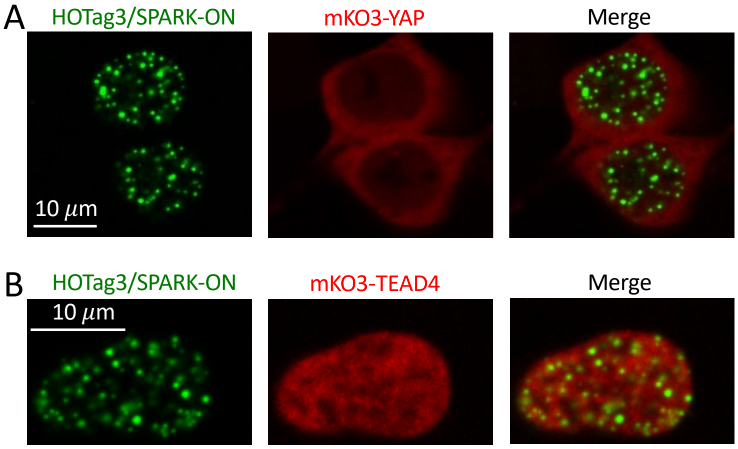

Fig. S14. HOTag3/SPARK-ON condensates do not contain YAP or TEAD. HEK293 cells transfected with HOTag3/SPARK-ON (CEL-NLS-mEGFP-HOTag3 + ZIF-NLS-EGFP(Y66F)-HOTag6). The cells were co-transfected with YAP or TEAD4 that is tagged with mKO3. The cells were treated with 1μM lenalidomide to induce SPARK-ON condensate formation.

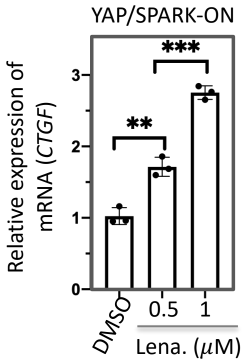

Fig. S15. Quantitative control of the YAP activity by the lenalidomide-activatable SPARK-ON. Data are mean ± SD (n = 3). ** P-value < 0.01.*** P-value < 0.001.

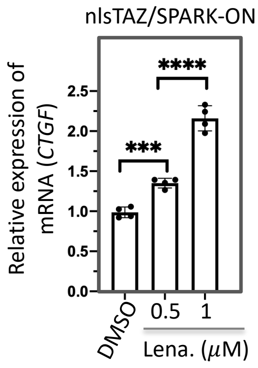

Fig. S16. Quantitative control of the nlsTAZ activity by the lenalidomide-activatable SPARK-ON. Data are mean ± SD (n = 3). *** P-value < 0.001.****P-value < 0.0001.

Table S1. List of all constructs.

Vector	Constructs or name of tools	Notes
pcDNA3.1	3xFlag-CRBN	3xFlag: DYKDHDGDYKDHDIDYKDDDDK
pcDNA3.1	3xFlag-CEL	CEL: c-terminal domain of CRBN (109aa)
pcDNA3	FLAG-DDB1	Addgene plasmid # 19918
pcDNA3.1	CEL-IFP2-SOScat	SOScat: catalytic domain of SOS2
pcDNA3.1	ZIF-RFP-CAAX	ZIF: zinc finger domain 2 of IKZF1 (31aa); RFP: mCherry
pENTR	ERK-KTR Clover	Addgene plasmid # 59138
pcDNA3.1	ERK-SPARK	Addgene plasmid #106921
pcDNA3.1	CEL-EGFP-HOTag3	EGFP: enhanced GFP
pcDNA3.1	CEL-Frb-EGFP-HOTag3	
pcDNA3.1	ZIF-EGFP-HOTag6	
pcDNA3.1	ZIF-NLS-EGFP*-HOTag6	EGFP*: non-green fluorescent mutant of EGFP with Y66F mutation
pcDNA3.1	CEL-EGFP-YAP	
pcDNA3	CEL-NLS-EGFP-TAZ	NLS: PAAKRVKLD
pcDNA3.1	IFP2-G3BP1	
pcDNA3.1	FKBP-IFP2-G3BP1	
pcDNA3.1	FUS-mKO3	mKO3: a red fluorescent protein, mKO2(M176F)
pcDNA3.1	FUS-mKO3-FKBP	
pcDNA3.1	mKO3-TIA1	
pcDNA3.1	FKBP-mKO3-TIA1	
pcDNA3.1	mKO3-TEAD4	
pcDNA3.1	mKO3-YAP	
pcDNA3.1	NPM1-EGFP	
pHR_SFFV	ZIF-NLS-EGFP*-HOTag6-T2A-CEL-EGFP-YAP	Lentiviral vector for constitutive expression of genes (SFFV promoter) T2A: self-cleaving peptide (EGRGSLL TCGDVEENPGP)
pHR_SFFV	CEL-NLS-EGFP-YAP	
pHR_SFFV	CEL-NLS-EGFP-TAZ	
pHR_SFFV	ZIF-NLS-EGFP*-HOTag6	
pHR_SFFV	ZIF-NLS-EGFP*	

Movie legend:

Movie S1. Lenalidomide-induced translocation of SOScat from cytosol to the plasma membrane. HEK293 cells were transfected with CEL-IFP2-SOScat and ZIF-RFP-CAAX. These cells were treated with lenalidomide. Left, IFP2 channel. Right, merged channel of IFP2 and RFP.

Movie S2. Activation of ERK upon lenalidomide-induced translocation of SOScat. HEK293 cells were transfected with ERK-SPARK, CEL-IFP2-SOScat and ZIF-RFP-CAAX. The cells were treated with lenalidomide.

Movie S3. ERK was not activated by DMSO. HEK293 cells were transfected with ERK-SPARK, CEL-IFP2-SOScat and ZIF-RFP-CAAX. The cells were treated with DMSO.

Movie S4. ERK was not activated by lenalidomide itself without ZIF. HEK293 cells were transfected with ERK-SPARK, CEL-IFP2-SOScat and RFP-CAAX. The cells were treated with lenalidomide.

Movie S5. Time-lapse imaging of the zoom-in area corresponding to Fig. 2B, showing initiation, growth and fusion of protein droplets. HEK293 cells expressing HOTag3/SPARK-ON were treated with lenalidomide.

Movie S6. Time-lapse imaging showing fusion and coalescence of protein droplets. HEK293 cells expressing HOTag3/SPARK-ON were treated with lenalidomide.

Movie S7. Time-lapse imaging showing disassembly of protein droplets upon removal of lenalidomide. HEK293 cells expressing HOTag3/SPARK-ON were preincubated with lenalidomide. Then lenalidomide was removed, followed by time-lapse imaging.

Movie S8. Time-lapse imaging showing phase separation of G3BP1 upon addition of rapamycin, and subsequent recruitment of FUS. HEK293 cells were transfected with SparkDrop-Frb (CEL-Frb-EGFP-HOTag3 and ZIF-EGFP-HOTag6), FKBP-IFP2-G3BP1 and mKO3-FUS. The cells were preincubated with 1 μM lenalidomide to form droplets of SparkDrop-Frb, followed by addition of 30 nM rapamycin.
